# *In Situ* Optical and X-ray Spectroscopy Reveals Evolution toward Mature CdSe Nanoplatelets by Synergetic Action of Myristate and Acetate Ligands

**DOI:** 10.1021/jacs.2c00423

**Published:** 2022-04-28

**Authors:** Johanna C. van der Bok, P. Tim Prins, Federico Montanarella, D. Nicolette Maaskant, Floor A. Brzesowsky, Maaike M. van der Sluijs, Bastiaan B. V. Salzmann, Freddy T. Rabouw, Andrei V. Petukhov, Celso De Mello Donega, Daniel Vanmaekelbergh, Andries Meijerink

**Affiliations:** † Debye Institute for Nanomaterials Science, Utrecht University, CS Utrecht 3584, The Netherlands; ‡ Laboratory of Physical Chemistry, Eindhoven University of Technology, AZ Eindhoven 5612, The Netherlands

## Abstract

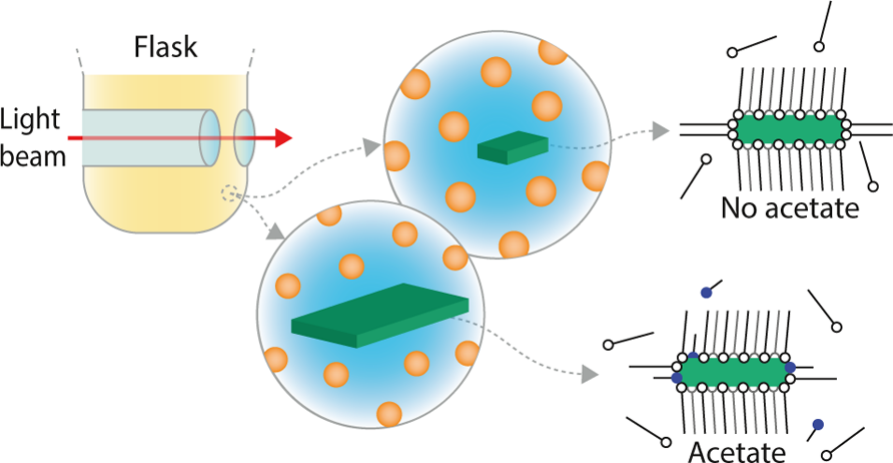

The growth of two-dimensional platelets of the CdX family (X = S, Se, or Te) in an organic solvent requires the presence of both long- and short-chain ligands. This results in nanoplatelets of atomically precise thickness and long-chain ligand-stabilized Cd top and bottom surfaces. The platelets show a bright and spectrally pure luminescence. Despite the enormous interest in CdX platelets for optoelectronics, the growth mechanism is not fully understood. Riedinger *et al.* studied the reaction without a solvent and showed the favorable role for short-chain carboxylates for growth in two dimensions. Their model, based on the total energy of island nucleation, shows favored side facet growth *versus* growth on the top and bottom surfaces. However, several aspects of the synthesis under realistic conditions are not yet understood: Why are both short- and long-chain ligands required to obtain platelets? Why does the synthesis result in both isotropic nanocrystals and platelets? At which stage of the reaction is there bifurcation between isotropic and 2D growth? Here, we report an *in situ* study of the CdSe nanoplatelet reaction under practical synthesis conditions. We show that without short-chain ligands, both isotropic and mini-nanoplatelets form in the early stage of the process. However, most remaining precursors are consumed in isotropic growth. Addition of acetate induces a dramatic shift toward nearly exclusive 2D growth of already existing mini-nanoplatelets. Hence, although myristate stabilizes mini-nanoplatelets, mature nanoplatelets only grow by a subtle interplay between myristate and acetate, the latter catalyzes fast lateral growth of the side facets of the mini-nanoplatelets.

## Introduction

Over the past decades, CdSe nanoparticles with a wide variety of shapes have been synthesized, for instance, quantum dots (QDs),^1^ nanorods,^2^ and nanoplatelets (NPLs). The latter family is of particular interest because CdSe NPLs exhibit by far the narrowest-band emission of them all.^3^ This remarkable property makes CdSe NPLs of interest for implementation in displays, as narrow-band emitters are needed to achieve higher energy efficiency and a wider color gamut.^4,5^ The narrow-band emission arises from the atomically accurate thickness of these quasi-two-dimensional nanoparticles resulting in strongly reduced inhomogeneous broadening of the emission spectra compared to QDs and nanorods.^6−8^

Implementation of NPLs in applications is only viable if high-quality NPLs are synthesized with high yield, and post-synthesis purification steps are minimized. This can only be achieved if the formation of NPLs proceeds with a higher yield than is currently obtained with state-of-the-art synthesis protocols. The synthesis protocol typically used for CdSe NPLs is a solution-based method, similar to the first reported method,^9^ where cadmium myristate and elemental selenium are heated in octadecene, and cadmium acetate is introduced in the reaction mixture at elevated temperature. Both CdSe QDs and NPLs form and must be separated by size-selective precipitation of the NPLs at a later stage.^10−15^ This is often not clear from reports on the synthesis and optical properties of CdSe NPLs. The NPL yield, if reported, is low; Moreels *et al.* reported an increase in yield for 3.5 ML NPLs using an alternative propionic acid-based method, while the authors reported a chemical yield of 40% when they used the standard method of Ithurria.^15^ Moreover, we find that with the standard CdSe NPL synthesis, the concentration of NPLs formed is far below that of QDs. Platelets are thus formed as a side product, and the low reaction yield and necessity of a purification step form a severe drawback for commercial application.

An improved synthesis method can resolve these issues but requires a better understanding of the reaction mechanism. Even though the first reports on the syntheses of zinc blende CdSe NPLs date from 2008,^9,16^ the formation mechanism of these NPLs is still under debate. Mechanisms proposed for zinc blende CdSe NPL formation include oriented attachment,^17−19^ templated growth,^20^ and continuous lateral growth.^10,12,21^ Because experimental data on nanocrystal nucleation and the evolution of (an)isotropic growth under realistic synthesis conditions are lacking, consensus on the formation mechanism has not yet been established. In addition, it is not clear why both long-chain and short-chain ligands are imperative for NPL formation, what controls the growth of both isotropic QDs and 2D NPLs, and how acetate catalyzes the formation of large NPLs in the widely used CdSe NPL synthesis method pioneered by Itthuria and Dubertret.^9^ In this study, we provide answers to these questions by studying CdSe QD and NPL nucleation and growth under realistic synthesis conditions.

It is challenging to study nanocrystal growth under reaction conditions that mimic the standard laboratory synthesis, and this has, so far, never been reported for CdSe NPLs. Previous studies on the growth of NPLs have either used *ex situ* analysis of aliquots taken during the reaction or *in situ* probing of the synthesis performed in capillaries.^12,17,21^ The pitfalls of the first approach are the low temporal resolution and disturbance of the reaction by taking aliquots. Furthermore, aliquots are not fully representative of the reaction mixture, and collecting quantitative aliquots is challenging. The second approach suffers from higher size polydispersity than for a synthesis performed in a flask due to insufficient mixing of reagents and temperature inhomogeneity. Additionally, the difference in reaction volume and diffusion rates in a capillary compared to a reaction flask can influence the reaction. Moreover, and this is crucial for the CdSe NPL synthesis, no additional reactants can be introduced during the reaction when using capillaries.

**Figure 1:**
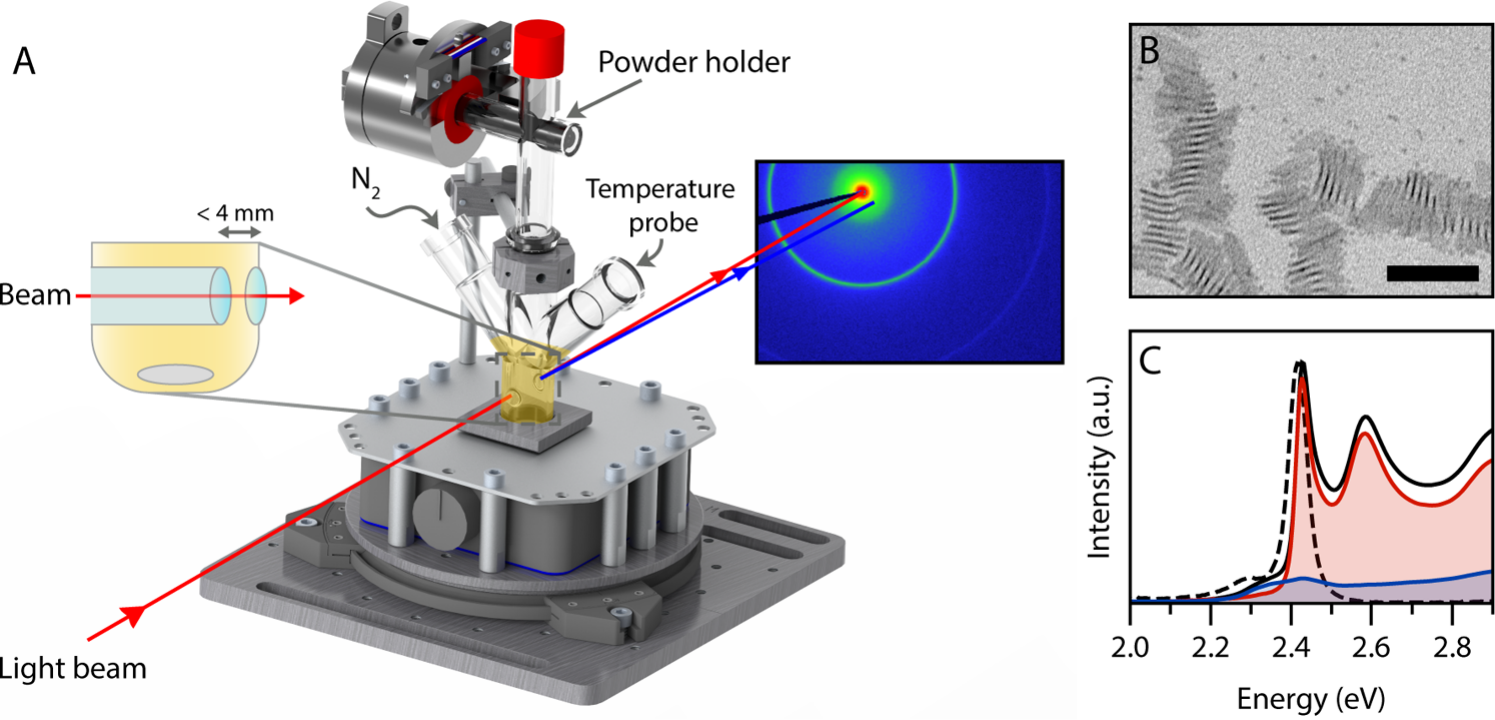
(A) Schematic of the experimental setup for *in situ* absorption spectroscopy and X-ray scattering experiments, containing a magnetic stirrer, a custom-made flask, and a powder injector. A protective container and heating ribbon are omitted from the image for clarity. A Teflon rod with a small cavity functions as a powder holder. This rod can be rotated remotely, upon which the powder falls into the reaction mixture. The flask can be connected to a nitrogen outlet. The reaction mixture is probed with either a collimated X-ray beam or UV/Vis light beam. An indentation in the reaction flask reduces the pathlength to less than 4 mm. (B) TEM image of the product obtained during the *in situ* SAXS experiment with the addition of cadmium acetate at 220 °C. Both NPLs and QDs are formed. The NPLs agglomerate into long stacks. The scalebar represents 50 nm. (C) Room-temperature absorption (solid) and emission (dashed) spectra of the same product confirm that both NPLs (2.43 eV) and QDs (∼2.3 eV) are formed during the reaction. The contribution of the NPLs and QDs is shown in red and blue, respectively. These spectra of the separate contributions were obtained after size-selective precipitation.

To resolve these issues and to allow *in situ* probing of the NPL growth, we used a specially designed three-neck flask adapted from a design in the literature with an indentation in the glass (Figure 1A).^22,23^ This indentation enables *in situ* UV/Vis absorption spectroscopy and small-angle X-ray scattering (SAXS) studies because it reduces the pathlength from the entire flask (leading to saturation of absorption) to a few millimeters. Hence, the intensity of transmitted light and X-rays is sufficient to conduct meaningful spectroscopy and scattering experiments. The use of this adapted three-neck flask also enables studies under standard reaction conditions, that is, inert atmosphere, high temperatures, and sufficient stirring, identical to the conditions used in the practical synthesis of CdSe NPLs. Moreover, a powder or liquid injector can be installed on top of the flask to remotely add other precursors during the synthesis. Therefore, no adaptions need to be made to the synthesis method for CdSe NPLs, where cadmium acetate is added at elevated temperatures. The growth of high-quality CdSe QDs has previously been investigated for a hot-injection synthesis demonstrating the unique capabilities of this home-built setup for *in situ* monitoring of nanocrystal formation.^24^

Here, we first follow and quantify the formation of CdSe NPLs and QDs and show that the currently used synthesis method yields CdSe NPLs in a much lower concentration with respect to CdSe QDs. A separation step is required to obtain nearly homogeneous solutions of NPLs, and the chemical yield is low, typically less than 50%. Then, we report on *in situ* UV/Vis absorption spectroscopy and SAXS measurements to monitor and quantify the formation of CdSe NPLs and QDs under different synthesis conditions with and without addition of acetate. By combining the results from both techniques, insights into the growth mechanism and the role of the ligands are obtained. Oriented attachment and lateral extension at the expense of QDs could be excluded as a formation mechanism. Our results show that both myristate and acetate play a pivotal role in the formation of NPLs. In the presence of long myristate ligands, a small concentration of mini-CdSe NPLs nucleates in addition to QDs, even without addition of acetate. In the continued absence of acetate, isotropic growth of QDs dominates. However, the addition of cadmium acetate triggers fast anisotropic growth of the mini-NPLs along the side facets which almost completely outcompetes further QD growth. The results are explained by a subtle interplay between long-chain myristate and short-chain acetate ligands in the formation and growth of QDs and NPLs. These insights can help adapt the synthesis to better control the interplay between ligands and favor the nucleation and growth of 2D NPLs to improve the yield of CdSe NPLs.

## Results and Discussion

The formation of NPLs was followed *ex situ* and *in situ* for the widely used synthesis method^9^ for CdSe NPLs which is described in detail in the Experimental Section. Briefly, it involves a solution-based reaction by heating Cd myristate and elemental Se in octadecene (ODE) typically to 240 °C with the addition of Cd acetate at a specific temperature. The home-built experimental setup for *in situ* measurements is depicted in Figure 1. The setup allows for the addition of cadmium acetate at any time and temperature during the synthesis without opening the reaction vessel. First, the optimal addition temperature was determined by adding cadmium acetate at 190, 220, or 240 °C. All experiments were performed in duplicate to verify reproducibility (Figure S1). The addition of cadmium acetate at 220 °C resulted in the formation of QDs (3.4 nm diameter^25^) and monodisperse 4.5 monolayer (ML) NPLs. Other temperatures yielded more than one NPL population or a higher fraction of QDs, as can be deduced from the *ex situ* absorption (Supporting Information S1.3). For this reason, we focus here on the CdSe NPL synthesis with the addition of cadmium acetate at 220 °C.

A transmission electron microscopy (TEM) image of the product using these reaction conditions (Figure 1B) shows the presence of both NPLs and QDs in the final product. The NPLs have a rectangular shape, typical for when anhydrous cadmium acetate is used,^26^ with lateral dimensions of 27 ± 2.2 by 7.5 ± 1.2 nm (Figures 1B and S3B). The NPLs tend to form large stacks. *Ex situ* absorption and emission spectra at room temperature are shown in Figure 1C. The contribution of the NPLs is highlighted in red and features the characteristic heavy- and light-hole transitions of 4.5 ML NPLs at ∼2.4 eV (∼510 nm) and ∼2.6 eV (∼480 nm).^9^ The QDs produce a relatively weak absorption and emission feature near 2.3 eV. The contribution of the QDs to the total absorption is indicated in blue.

The fractions of QDs and NPLs in the absorption spectrum were determined with the absorption spectra obtained after size-selective precipitation. From this, we estimated (Supporting Information, Section S1.4) concentrations of QDs and NPLs in the reaction mixture of 2.9 and 0.35 μM, respectively. This is a rough estimate because the QD fraction still contains residual NPL absorption. Furthermore, the QD concentration is underestimated by the scattering of stacked NPLs at 2.35 eV. This scattering also affects the absorbance at 300 nm. Nevertheless, this rough estimate reveals that the NPL concentration is an order of magnitude lower than the QD concentration. This may seem surprising considering the weak absorption feature of the QDs in Figure 1C, but it is a consequence of the much lower QD extinction coefficient due to the difference in the volume (21 nm^3^ for the QDs compared to 263 nm^3^ for the NPLs) and intrinsic absorption coefficient μ_i_ (2 × 10^5^ cm^–1^ compared to 6 × 10^5^ cm^–1^) at a wavelength of 300 nm.^13,27,28^ Note that even though the concentration of NPLs is about 10 times lower, the CdSe yield for the QDs and NPLs is similar due to the large volume difference. The difficulty in extracting the absolute concentrations and the relatively low NPL concentration stresses the importance of exploring the reaction mechanism using *in situ* studies.

### *In Situ* Study of the Evolution of the Reaction

**Figure 2:**
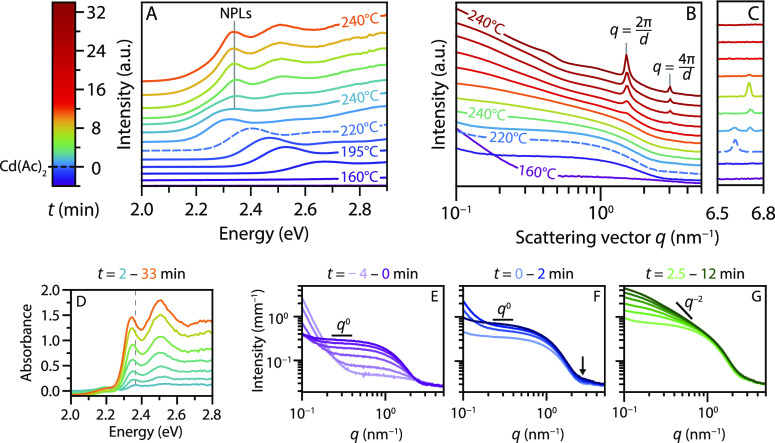
Absorption spectra (A) and scattering patterns (B,C), shifted for clarity, obtained *in situ* during the synthesis of CdSe NPLs. The colors of all curves correspond to the times indicated in the legend. Temperature increases from ∼160 to 240 °C and is then kept at 240 °C. Cadmium acetate is added at 220 °C (dashed line, *t* = 0 min). Blue to cyan absorption spectra show growth of QDs. The absorption features of the NPLs (2.35 eV) become visible shortly after addition of the acetate. The SAXS data also indicate growth of isotropic particles (purple to blue, scattering intensity scaling as *q*^0^), followed by growth of NPLs after addition of acetate (blue to red, *q*^0^ regime disappears). Structure factor peaks are observed due to stacking of the formed NPLs (*d* = 2π/*q* = 4.2 nm). In C, the atomic scattering peak of solid cadmium acetate can be observed. The peak shifts around 230 °C, probably due to a change in the crystal structure. After ∼10 min at 240 °C, the acetate is completely dissolved. (D) The QD absorbance (a spectrum at 230 °C) is subtracted from the data in A. The resulting spectra show the heavy- and light-hole transition of 4.5 ML NPLs at 2.35 and 2.52 eV, respectively, shifted to lower energies compared to room temperature due to temperature effects (see main text). A dashed gray line is added to emphasize the shift of the absorption maximum in the first few frames due to quantum confinement in the lateral dimensions. (E) SAXS patterns until addition of the acetate. (F) SAXS patterns shortly after addition of acetate. The scattering increases at *q* = 2–3 nm^–1^, but still, a *q*^0^ regime is observed. (G) SAXS patterns 2.5–12 min after the acetate addition, reflecting particle growth at 240 °C. The slope at *q* < 1 nm^–1^ becomes steeper than the previous *q*^0^ scaling, indicating growth of anisotropic particles.

The temperature and time evolution of the absorption spectra and SAXS data of the *in situ* measurements is shown in Figure 2A,B. The colors reflect the time relative to the cadmium acetate addition at *t* = 0 min, as is specified by the legend on the left. Both data sets show the formation of QDs starting from ∼170 °C (dark blue) by the increase in UV absorption and scattering. The *q*^0^ slope extending to 1 nm^–1^ and shape of the early X-ray scattering patterns match the form factor of spherical particles (Figure S8 in S2.3.1), that is, the QDs. These QDs grow over time shifting the absorption maximum to lower energies and slightly shifting the scattering minimum to smaller *q* values (compare the dark blue and dashed scattering pattern around *q* = 3 nm^–1^). These features are consistent with the theoretical scattering patterns for growing QDs, but a minor contribution of small anisotropic nanostructures cannot be excluded.

The evolution of the QD growth in the scattering patterns is shown in more detail in Figure 2E. A shallow minimum is visible at *q* ∼ 3 nm^–1^ and shifts to ∼2.8 nm^–1^ over time. The minimum is shallow because of the polydispersity in size. The intense scattering at *q* < 0.2 nm^–1^ in the first few frames is caused by the scattering of undissolved μm-large selenium particles (Figure S6F). The additional scattering reduces when the selenium dissolves and is not significant anymore at *t* ∼ 0 min. No lamellar phase is observed in the SAXS data when nanoparticles start to form. The cadmium myristate dissolves around 100 °C (Figure S6A), well before the onset of nucleation. This rules out templated NPL growth on a lamellar Cd myristate phase and is consistent with other reports.^10,21,29^

The absorption spectra in Figure 2A show that after the addition of cadmium acetate at 220 °C (dashed), the existing QDs continue to increase in size over the 1st minute (*i.e.*, the absorption feature shifts to lower energy). Within 1 minute after the acetate addition, a new absorption feature appears at 2.35 eV, quickly outgrowing the QD absorption. In the scattering data, the growth of small particles is apparent from the increase in intensity at *q* = 2–3 nm^–1^ (Figure 2F, arrow). The slope at *q* < 1 nm^–1^ still scales with *q*^0^, typical for isotropic particles. After several minutes (Figure 2B, yellow and orange), the slope starts to deviate from *q*^0^ which shows that anisotropic particles have formed. The slope is not equal to a *q*^–2^ slope expected for 2D materials (Figure 2G) because the scattering pattern originates from QD and NPL scattering resulting in a slope between *q*^0^ and *q*^–2^.

To monitor the growth of the NPLs, the QD contribution was subtracted from the absorption spectra. The resulting spectra are shown in Figure 2D and clearly contain the heavy- and light-hole transitions characteristic of 4.5 ML NPLs (at 2.35 and 2.52 eV, respectively). Note that at elevated temperatures, the peak position shifts to lower energies, and the peak width increases compared to room temperature (Figure 1C). The peak maximum shifted from 2.43 eV at room temperature to 2.35 eV at 240 °C, corresponding to a shift of 0.37 meV/°C, which matches values of 0.31–0.44 meV/°C reported for the emission of 4.5 ML NPLs.^30,31^

The intensity of the NPL absorption in Figure 2D increases over time. In addition, the absorption maximum shifts noticeably between the first few displayed spectra (light blue to green), more than expected from temperature effects. Both the intensity increase and shift in the position are evidence of the growth in the NPL lateral dimensions.^32^ At first, the still laterally small NPL dimensions lead to three-dimensional confinement of the exciton. As the NPLs grow, the lateral dimensions quickly exceed the confinement regime, and consequently, the peak does not shift any further. The small fluctuations of the absorption maxima (green to orange) are caused by temperature fluctuations, varying between 235 and 245 °C.

Overall, the evolution of absorption spectra in the different synthesis stages is consistent with the scattering data. Additionally, the scattering data show the stacking of NPLs after ∼12 min as structure factor peaks begin to appear at *q* = 1.5 nm^–1^ and *q* = 3.0 nm^–1^.^33^ These *q*-values are consistent with linear stacks of NPLs with a center-to-center distance of *d* = 2π*n*/*q* = 4.2 nm (with *n* = 1 or 2). This distance is set by twice the length of the myristate ligand plus the thickness of a 4.5 ML NPL (1.3 nm).^34^ Thus, the length determined for the myristate ligands is 1.45 nm, slightly shorter than the 1.7 nm expected for myristate with a fully extended carbon chain.^35^ This means that the ligands are not fully extended, or they slightly interpenetrate.

The scattering data further reveal the presence and slow dissolution of solid cadmium acetate crystallites. The reappearance of strong scattering at *q* < 0.2 nm^–1^ at *t* = 0 min in Figure 2E and the appearance of a peak in the scattering pattern at *q* = 6.63 nm^–1^ (Figure 2C) indicate the presence of undissolved cadmium acetate crystallites (Figure S6E). The peak’s intensity decreases at 230 °C, and a new peak appears at *q* = 6.71 nm^–1^. This shift is probably caused by a change of the cadmium acetate crystal structure.^36,37^ The signal disappears completely after ∼10 min at 240 °C. Similar behavior is observed when cadmium myristate and cadmium acetate are heated without the presence of selenium (Figure S6D). When cadmium acetate is heated in the absence of cadmium myristate, the peak in Figure 2C does not disappear until a temperature of 255 °C (Figure S6E), that is, the melting temperature of cadmium acetate.^36^ We conclude that the dissolution of cadmium acetate is assisted by reaction with cadmium myristate forming Cd(Ac)_2–*x*_(Myr)_*x*_. These results show that NPLs start forming when cadmium acetate is still, at least partly, present as a solid (compare lemon-colored lines in Figure 2A,D with that in 2C).

**Figure 3:**
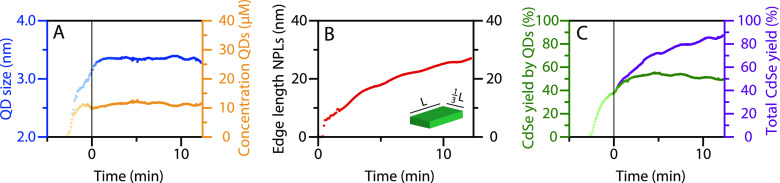
Fit results extracted from the SAXS patterns in Figure 2B. (A) QD diameter (blue) and QD concentration (orange). (B) Length *L* of the longest edge of the NPLs. The aspect ratio of the lateral dimensions is 1:3. *L* could be underestimated in the first few minutes because the concentration of the NPLs was kept at a constant value of 0.6 μM during the fitting procedure. (C) Percentage of CdSe consumed by the formed QDs in green (CdSe yield by QDs) and by the QDs and NPLs in purple (Total CdSe yield).

The size, aspect ratio, and concentration of the QDs and NPLs can be extracted by fitting the SAXS data in Figure 2B; this makes it possible to follow the formation of QDs and NPLs over time. The scattering patterns were carefully corrected for background effects and analyzed to obtain information on the size and shape evolution of nanostructures in the reaction mixture, as described in detail in the Supporting Information. This analysis gave an NPL aspect ratio of 1:3 and a concentration of 0.6 μM. The evolution of size and concentration for the QDs is shown in Figure 3A in blue and orange, respectively. The length of the largest lateral dimension *L* of the NPLs is shown in Figure 3B in red. The scattering patterns were fitted until the NPLs started to stack at *t* = 12 min. The oscillation in the data is caused by a temperature fluctuation during the synthesis.

Figure 3A shows that the QD concentration quickly increases to ∼11 μM during the heat-up from 170 to 220 °C and remains constant afterward. The QD diameter increases during this period as well. In the first 1.5 min following cadmium acetate addition, the QDs continue growing from 3.16 to 3.36 nm, consistent with the analysis of the absorption spectra discussed earlier. After 1.5 min, the QD growth stops.

Immediately after the cadmium acetate addition, the lateral dimensions of the NPLs grow, as shown in Figure 3B. Within half a minute after the addition of cadmium acetate, NPLs with lateral dimensions of 5.0 by 1.7 nm are visible. This indicates that small NPLs are present before the acetate addition (*vide infra*) but not easily observed because the scattering is negligible compared to the QD scattering (Figure S9B). The edge lengths rapidly increase after acetate addition to 26 by 8.7 nm after 12 min, just before the NPLs start to stack. TEM analysis gives lateral dimensions of 27 ± 2.2 by 7.5 ± 1.2 nm (Figures 1B and S3B), which matches the SAXS results and confirms the reliability of the fitting procedure.

The constant concentration of QDs after the cadmium acetate addition indicates that NPL formation is not due to attachment of seed QDs. Moreover, the diameter of the QDs is already 3.16 nm when cadmium acetate is added, by far exceeding the NPL thickness of 1.3 nm. The constant QD radius and concentration show that the growing NPLs do not consume existing QDs, but consume the CdSe monomers that are still present in the reaction mixture. The CdSe yield of the reaction, calculated from the incorporated amount of selenium in the nanoparticles (S4.1) with respect to the selenium added in the reaction mixture, as shown in Figure 3C supports this. The available CdSe units are far from depleted at *t* = 0 min: the total yield (purple) is only 40%. Over the 12 min following cadmium acetate addition, an additional 10% of the available CdSe is incorporated in the QDs. Simultaneously, 40% of the CdSe is incorporated in the NPLs. Hence, the growth of the NPLs is much faster than that of the QDs after the addition of cadmium acetate.

Additionally, the 10% increase in yield due to QD growth indicates that the QDs do not dissolve in favor of NPL growth. The QDs are an undesired byproduct of the reaction and consume roughly half of the available precursors. The concentration of QDs (∼11 μM) is much higher than the NPL concentration (0.6 μM), as was also estimated from the *ex situ* absorption spectrum. Although most QDs formed before cadmium acetate addition induced the growth of NPLs, they still form when cadmium acetate is added much earlier (Figure S3A), and such procedures produce 3.5 ML NPLs instead of 4.5 ML NPLs.

### *In Situ* Study without Cadmium Acetate

**Figure 4:**
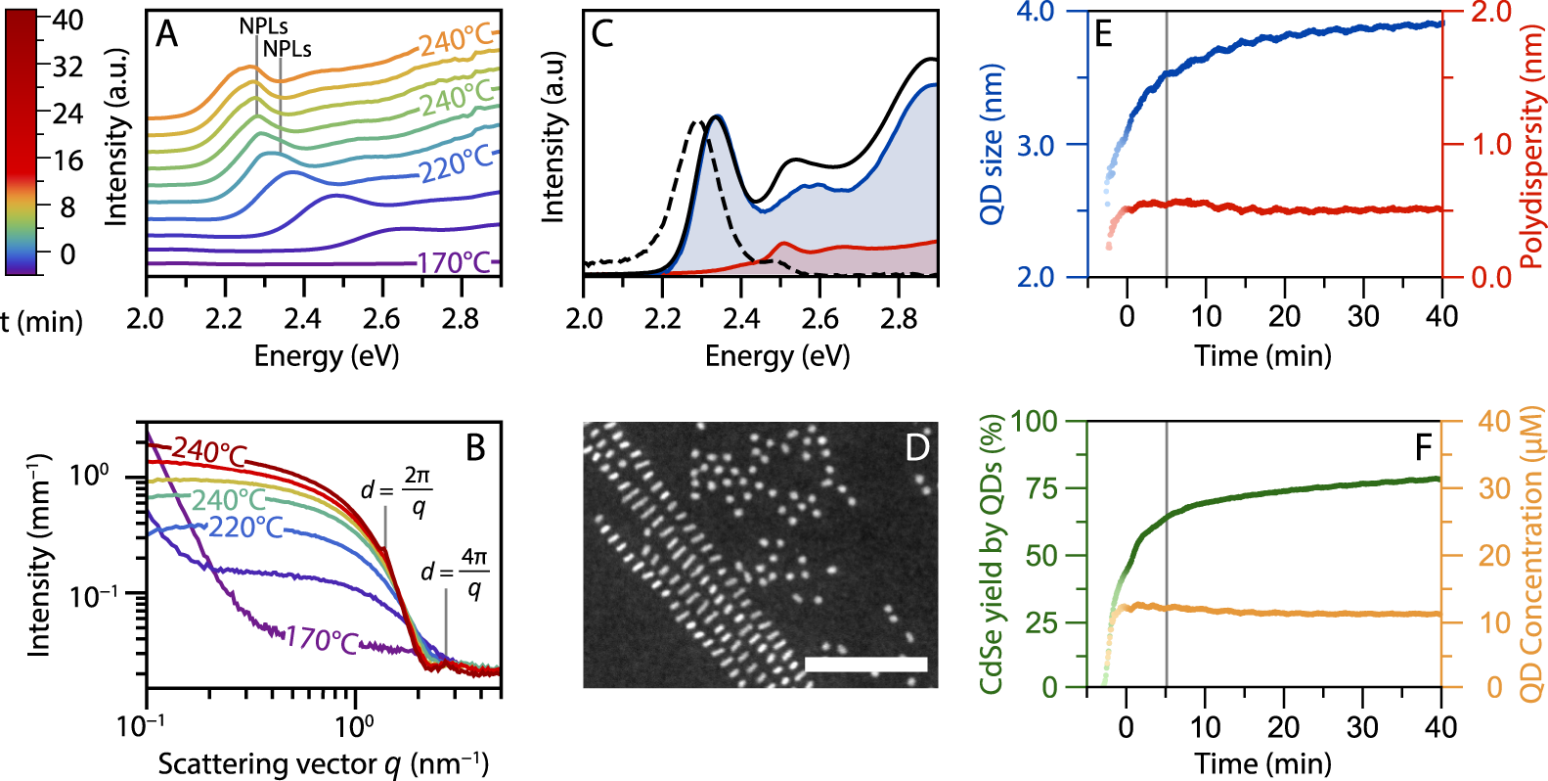
Absorption spectra (A) and scattering patterns (B) of *in situ* experiments when no acetate is added to the reaction mixture. In (A), the spectra are shifted in intensity for clarity. The weak absorption features of 4.5 mini-NPLs at 2.34 eV and 5.5 ML mini-NPLs at 2.28 eV are labeled with NPLs for clarity. Features shift to lower energies compared to room temperature due to temperature effects. The structure factor peaks of the stacked 5.5 ML mini-NPLs are labeled in (B) (*d* = 4.5 nm). (C) Absorption (solid) and emission (dashed) spectra at room temperature of the product obtained during the *in situ* SAXS experiment. Next to QD absorption and emission (2.3 eV), also a second population of nanoparticles is present: mini-NPLs (2.47 eV). The blue and red curves represent contributions from the supernatant (QDs, blue) and precipitate (predominantly mini-NPLs, red) after selective precipitation. (D) HDAAF-STEM image of the reaction product showing stacked mini-NPLs and QDs, scalebar 50 nm. (E) Diameter (blue) and polydispersity (red) of the QDs extracted from fitting the SAXS data in (B). (F) QD concentration during the reaction (orange) and the reaction yield (green). This yield only accounts for the CdSe consumed by the QDs. The total yield, including the mini-NPLs, is ∼3% higher than the yield in (F). The gray line in (E) and (F) at ∼5 min indicates when the stacking of the mini-NPLs starts to contribute to the total scattering.

To study the role of cadmium acetate further, we compare the results discussed above with a synthesis using the same reaction conditions but without the addition of cadmium acetate. The *in situ* absorption spectra and SAXS patterns are shown in Figure 4A,B, respectively. They show, up until 220 °C, a similar QD evolution compared to the results in Figure 2A,B. In contrast to the experiment of Figure 2, we add no acetate at 220 °C at *t* = 0. Nevertheless, a new absorption feature still arises around *t* = 2 min. The feature, labeled with “NPLs” in Figure 4A, is first visible at 2.34 eV (light blue) and later shifts to 2.28 eV (green). Clearly, a mixture of two types of nanoparticles still forms in the absence of acetate (S3).

The presence of two populations of nanoparticles is evident in the *ex situ* absorption and emission spectra as well (Figure 4C). The luminescence and absorption spectra at room temperature show, in addition to the QD absorption and emission, a peak of a second population of nanoparticles at 2.47 eV. These two populations could be separated with size-selective precipitation. The high-angle annular dark-field scanning transmission electron microscopy (HAADF-STEM) image in Figure 4D reveals that the two species of nanoparticles are spherical QDs and small anisotropic nanoparticles. The anisotropic nanoparticles appear as rod-like structures in the HAADF-STEM image with the largest lateral dimension equal to ∼5 nm. These nanoparticles stack during the synthesis as well and give rise to the structure factor peaks in the SAXS data at *q* = 1.5 nm^–1^ and *q* = 3 nm^–1^. These *q*-values are due to stacking at a center-to-center distance of 4.5 nm, which is larger than the center-to-center distance found above for the stacked 4.5 ML NPLs (Figure 2B) by 0.3 nm. This difference matches the thickness of one CdSe monolayer, that is, half a unit cell. The anisotropic nanoparticles are thus likely “mini-NPLs” of 5.5 ML thickness. They appear as rod-like structures on (S)TEM images and in a HDAAF-STEM tilt series (Figure S4) because the shortest lateral dimension is not much larger than the thickness of the mini-NPLs, when assuming a similar aspect ratio as that of the large NPLs (3:1).

Heating the reaction mixture to 240 °C results in the formation of 5.5 ML mini-NPLs. Mini-NPLs with a thickness of 4.5 ML can also be synthesized by lowering the final reaction temperature to 190 °C instead of 240 °C. Structure factor peaks at the same position as in Figure 2B were observed in SAXS data obtained using these reaction conditions (Figure S13). In the reaction with acetate, the 4.5 ML mini-NPLs rapidly grow to form 4.5 ML NPLs. Without acetate, 5.5 ML mini-NPLs form upon heating to 240 °C (Figure 2 and Figure S13). This temperature dependence suggests that the two absorption features in Figure 4A, labeled with NPLs, correspond to mini-NPLs with a thickness of 4.5 ML formed below 240 °C (2.34 eV, slightly higher energy than that of large 4.5 ML NPLs) and 5.5 ML mini-NPLs formed after a temperature of 240 °C is reached (2.28 eV). Note that the stronger confinement for the small lateral dimension of the 5.5 ML mini-NPLs results in stronger temperature dependence of the absorption maximum, shifting it to a higher energy (2.47 eV) at room temperature compared to the thinner but larger 4.5 ML NPLs (2.43 eV).

The size, polydispersity, and concentration of the QDs and mini-NPLs and the reaction yield were extracted from the SAXS data as well (Supporting Information S2.3.3).^38^ The form factor of a disk was used to approximate the mini-NPLs shape. An average radius for the mini-NPLs of 3.5 nm was obtained with 4.5% polydispersity and a concentration of ∼0.25 μM. The fit results for the QDs are shown in Figure 4E,F. The evolution of the QD concentration in the cases with and without cadmium acetate is very similar, (orange, Figures 3A and 4F). The polydispersities are also similar, reaching a value of ∼0.5 nm after an initial increase. However, the increase in QD size over time is strikingly different between the experiments. While acetate addition leads to stagnating QD growth shortly after *t* = 0 (Figure 2), the growth continues until a final size of 3.8 nm at *t* = 10 min in the absence of acetate. The 0.4 nm difference in final size shows that without cadmium acetate, more CdSe precursor is available for QD growth. In other words, the presence of cadmium acetate results in precursor consumption by the growth of the second population of nanocrystals, that is, the NPLs.

The same conclusion can be drawn by comparing the reaction yields with and without the addition of cadmium acetate. The yield of the reaction without cadmium acetate is given in Figure 4F in green. The yield in Figure 4F accounts only for the CdSe incorporated in the QDs and can therefore directly be compared to the corresponding results of the experiment with acetate (green in Figure 3C). Similar values for the yield are obtained until *t* = 0 min (38%). However, without the addition of cadmium acetate, the final CdSe consumption by QDs is much higher (70% compared to 50% after 12 min). Without acetate, the anisotropic particles (mini-NPLs) take up 3% of the total CdSe content (S4.2), compared to 40% CdSe incorporated in NPLs in the experiment with acetate. We verified the yield derived from the SAXS results by inductively coupled plasma-optical emission spectrometry (ICP-OES) analysis. Yields of 47 ± 1.5 and 75 ± 1% were obtained with ICP at reaction times corresponding to *t* = 10 min and *t* = 30 min in Figure 4F, respectively. This matches well with the SAXS results. The results from optical absorption, SAXS, and ICP-OES are consistent and show that the contribution of the stacked and individual mini-NPLs to the total yield for the synthesis without acetate is low.

We learn from these experiments that the addition of the acetate strongly enhances the growth rate of already existing anisotropic particles (mini-NPLs) but not the growth rate of the QDs. The presence of acetate promotes the lateral growth of already existing NPLs so strongly that almost no reactants are used in the further growth of the QDs. The question rises if acetate also affects the nucleation of mini-NPLs versus small QDs in the initial stage of the reaction. From the fit, we estimate a mini-NPL concentration of 0.25 μM without and an NPL concentration of 0.6 μM with acetate. The 0.25 μM is a lower limit as only the stacked mini-NPLs contribute, while the 0.6 μM is a more reliable estimate of the total concentration of NPLs. Both concentrations of (mini)-NPLs are very small compared to the QD concentration. Hence, acetate does not strongly affect the initial ratio between QDs and mini-NPLs, but its role is to favor very strongly the lateral growth of already existing mini-NPLs.

### Growth Mechanism of CdSe NPLs

**Figure 5:**
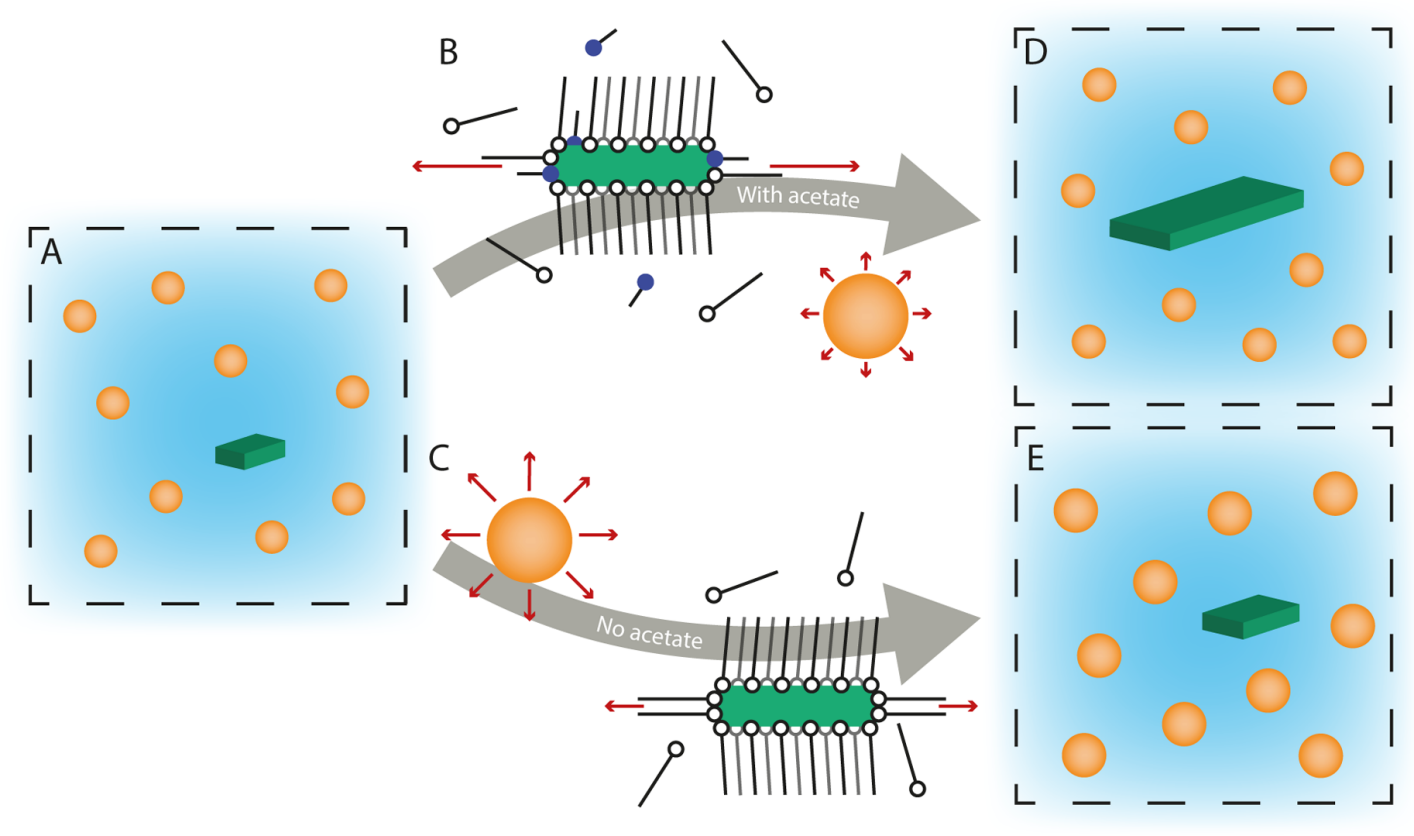
Schematic overview of the CdSe NPL growth mechanism. (A): Nanoparticles nucleate and grow. An order of magnitude more QDs are formed than NPLs. Middle (B): The addition of acetate results in faster monomer consumption. With the reaction conditions used here, most monomers are consumed by growth of the small facets of the NPL (red arrows). As a result, the NPLs grow laterally, and the QDs do not grow much (D). The concentration of QDs and NPLs is unaffected by the addition of an acetate. Middle (C): If no acetate is added, both QDs and mini-NPLs grow with a comparable monomer consumption (red arrows). This way the NPLs remain small, and slightly larger QDs are obtained compared to with the addition of acetate (E).

Figure 5 gives a schematic overview of the reaction mechanism based on the data discussed in this work. In the early stage of the reaction, both QDs and mini-NPLs with small lateral dimensions form. The mini-NPLs have an order of magnitude lower concentration than the QDs. Once formed, the mini-NPLs are likely stabilized by strong Van der Waals interactions between the long alkyl chains of the ligand layers on the top and bottom facets. The stability of the myristate layers on the large facets is evidenced by the center-to-center distances observed for (mini-)NPL stacks by *in situ* SAXS. Even at high temperature, these distances are consistent with spacing by slightly interpretating myristate layers. Furthermore, a higher concentration of long-chain ligands results in increased NPL absorption relative to QD absorption.^29^

When solid cadmium acetate crystallites are added to the reaction mixture, the solution concentration of cadmium acetate is initially low as the crystallites dissolve slowly. As the solution concentration of acetate increases, the monomer consumption by the small facets of the NPL starts to outcompete the monomer consumption by the QDs. Importantly, our work demonstrates that the conditions under which NPLs grow are not required to make NPLs nucleate. Indeed, we observe mini-NPLs even if we do not add acetate to the reaction (Figure 4) and at approximately the same final concentration. On the other hand, the formation of mini-NPLs seems to be disrupted if too much acetate is available too early in the reaction. For example, when cesium acetate (melting point 195 °C) instead of cadmium acetate is added at 190 °C while heating to 240 °C, only 3D particles are formed (Figure S5A). Under these conditions, the concentration of acetate in solution is too high immediately after addition. On the other hand, NPLs are formed when cesium acetate is added at 190 °C without further heating to 240 °C (Figure S5B). The results are in line with previous reports on acetates catalyzing NPL growth.^9^ All reported acetates have melting points well above 190 °C (see Supporting Information Section S1.9). Note that the addition of different acetates also affects the lateral shape of the NPLs. Likely other factors next to the melting point, such as the reactivity of the cation acetate, also affect the growth of the NPLs.

The model proposed by Norris *et al.*(10,39) explains anisotropic 2D growth of (mini-)NPLs by a lower activation energy for island nucleation on side facets compared to top and bottom facets. This activation energy is determined by the volume, area, and line energies of zinc blende CdSe. The synthesis condition used to construct the model, that is, NPLs formed in a melt of cadmium acetate and selenium, deviates significantly from that of the solution-based synthesis studied here. Furthermore, the work of Norris *et al.* considers constant reaction conditions, while in our and standard experiments, the conditions are changed midway by addition of cadmium acetate. The presence of acetate affecting the CdSe monomer consumption stresses the importance of including the effect of the surfactants on anisotropic growth.

The effect of cadmium acetate addition can be included in an NPL growth model as a drop in the values for area, line, and volume energies. Lowering any of these energies results in a decrease in the activation energy for “island nucleation”, that is, the formation of a new monolayer on an existing facet, and hence an acceleration of crystal growth. With the right combination of energy values, island nucleation rates on the narrow facets outpace that on the top and bottom facets. The fastest growing facets can consume available monomers so quickly that growth on other facets effectively stops. Interestingly, further lowering area, line, and volume energies decreases the difference in the activation energies between narrow and large facets. This explains why the addition of too much acetate early in the reaction results in isotropic growth, as it decreases the anisotropy in growth rates.

The middle part of Figure 5 gives a molecular picture of the potential effects of cadmium acetate on the volume, surface, and line energies. The volume energy may become more negative when cadmium acetate is present in the reaction mixture by lowering the solubility of the cadmium precursor. The surface and line energies decrease due to changes in the ligand coverage of the NPL by exchanges of myristate ligands with acetate. The reaction rate for all NPL and QD facets will likely increase. However, as the activation energy for island nucleation on narrow NPL facets is the lowest, the effective monomer consumption by the narrow facets of the NPL will outcompete the monomer consumption by the QDs and large facets (red arrows). A higher ligand exchange of the side facets by short acetate ligands, due to weaker binding sites at the edges,^11^ may further enhance the 2D growth by lowering the steric barrier for monomer attachment on side facets.

The formation of large NPLs, using the standard CdSe NPL reaction protocol, thus ultimately relies on the synergy between cadmium myristate and cadmium acetate ligands, where the cadmium myristate reduces isotropic growth by stabilizing mini-NPLs at the early stages of the reaction, and addition of cadmium acetate results in faster growth on the small side facets. The molecular picture that we present is in line with the model of Norris, which emphasizes that an appropriate balance of surface and line energies is required for two-dimensional growth. When no acetate is added, both the QDs and mini-NPLs grow with a comparable monomer consumption (Figure 5c). This can be deduced from the similar ratio between the concentrations and contributions to the total yield (11 μM compared to 0.25 μM and 75% yield and 3% yield after 12 min). The presence of acetate favors lateral growth of the platelets and, by precursor consumption, impedes further growth of the QDs. Acetate addition determines the sizes but not the final concentrations of the two types of particles (Figure 5d,e).

## Conclusions

With our home-built setup, we were able to probe *in situ* the formation of CdSe NPLs with (or without) the addition of cadmium acetate for the standard NPL synthesis under realistic reaction conditions. Analysis of *in situ* absorption and scattering experiments shows that both isotropic and anisotropic particles form at an early stage of the reaction even without short-chain ligands. NPLs with large lateral dimensions (∼27 by 7.5 nm) are formed due to a synergy between the long myristate ligands stabilizing top/bottom facets of the 2D structures and short acetate ligands that promote fast growth of the NPL side facets but do not affect the NPL concentration. The concentration of NPLs (∼0.6 μM) is low compared to the QDs (∼11 μM), which are always formed as a prominent and undesired side product. These QDs are responsible for a low NPL reaction yield and have to be removed using size-selective precipitation. The new insights in the mechanism of CdSe NPL formation can help improve the synthesis conditions (such as type and concentration of ligands, reaction temperature, etc.) to optimize the mini-NPL *versus* QD formation and NPL growth to improve the NPL yield.

## Experimental Section

4.5 ML CdSe NPLs were synthesized as reported by Ithurria *et al.*(12) For the *in situ* measurements, the synthesis was scaled down by a factor of 2. 85 mg of cadmium myristate (0.15 mmol, see the Supporting Information), 6.0 mg of elemental selenium (0.075 mmol), and 7.5 mL of ODE were loaded in a specially designed three-neck flask and degassed under vacuum at room temperature for 1 h. After degassing, the mixture was put under a nitrogen atmosphere, and the powder injector (Figure 1A) was connected with 26 mg of cadmium (0.11 mmol) acetate in the powder holder. The system was flushed three times by applying a vacuum or nitrogen flow. Then, the mixture was heated to 240 °C with a rate of 15 °C/min using a heating ribbon around the flask. For the *in situ* absorption measurements, a transparent medium was needed to align the flask with the UV/Vis light beam. Therefore, an additional heating step to ∼110 °C, before heating to 240 °C, was implemented to melt and dissolve the cadmium myristate.

A series of experiments was conducted to investigate the role of acetate in the formation of CdSe NPLs. In the various experiments, the flask with the reaction mixture was heated to 240 °C. Cadmium acetate was added at 190, 220, or 240 °C by rotating the powder holder. Furthermore, an experiment without the addition of acetate was performed. During the reaction, UV/Vis absorption spectra or X-ray scattering patterns were recorded. After heating up and a reaction time of 10–45 min at 240 °C, the mixture was let to cool down to room temperature. 0.5 mL of oleic acid was added at 70 °C and 7 mL of hexane at room temperature. The product was purified twice by adding 45 mL of a methanol/butanol mixture (1:2) and centrifuged at 3000 rpm (∼1000 RCF). Special care was taken to precipitate all the product to ensure that the absorption spectra give a reliable view of the ratio QDs-to-NPLs. If the supernatant was not transparent, more methanol was added. Finally, the product was redispersed in 4 mL of hexane.

The SAXS experiments were conducted at the SWING beamline of synchrotron Soleil at an energy of 16 keV and a sample-to-detector distance of 1.83 m. This allowed us to probe a ***q***-range of 0.05 nm^–1^–8 nm^–1^. 2D scattering patterns were recorded every 5 s with an exposure time of 3 s. The background scattering of the solvent, reactants, and flask was subtracted from the azimuthally integrated 2D scattering patterns (S2.1 and S2.2). Models for the fitting of the scattering patterns are discussed in the Supporting Information (S2.3).

The *in situ* absorption experiments were performed using a DH-2000-BAL lamp as the excitation source, 200 μm core solarization resistant fibers, and a USB4000 spectrometer all from Ocean Insight. To obtain a collimated light beam with a diameter smaller than 5 mm, that is, the diameter of the indentation in the glass, the following optics was used: 14 mm focal point (f14) VIS achromatic lens, 350 nm long pass filter, f30 VIS aspherical achromatic lens, 200 μm pinhole, and f14 VIS aspherical, achromatic lens. The light bundle after the sample was coupled into a fiber using a f14 VIS aspherical, achromatic lens. The lenses and filter were obtained from Edmund Optics. Spectra were recorded with an integration time of 100 ms. The absorbance was calculated afterward, taking an *I*_0_-spectrum just before nanoparticles started to form.

## Supporting Information Available

Chemicals, additional experimental methods, additional TEM images, temperature-dependent scattering data of solvents and precursors, correction and fitting procedure of SAXS data, ICP data, concentration determination using absorption data, and additional SAXS data (PDF)
