# Supplementary material for: In Situ Optical and X-ray Spectroscopy Reveals Evolution toward Mature CdSe Nanoplatelets by Synergetic Action of Myristate and Acetate Ligands
Source: J Am Chem Soc. 2022 Apr 28;144(18):8096–105. doi: 10.1021/jacs.2c00423 (PMC9100465; doi:10.1021/jacs.2c00423)
Supplement: Supplementary file 1 — ja2c00423_si_001.pdf [file ja2c00423_si_001.pdf]

Supporting Information

In Situ Optical and X-ray Spectroscopy Reveal Evolution Towards Mature CdSe Nanoplatelets by Synergetic Action of Myristate and Acetate Ligands

Johanna C. van der Bok[†,#], P. Tim Prins[†,#], Federico Montanarella[†], D. Nicolette Maaskant[†], Floor A. Brzesowsky[†], Maaike M. van der Sluijs[†], Bastiaan B. V. Salzmann[†], Freddy T. Rabouw[†], Andrei V. Petukhov[†§], Celso De Mello Donega[†], Daniel Vanmaekelbergh[†*] and, Andries Meijerink[†]

† Debye Institute for Nanomaterials Science, Utrecht University, 3584 CS Utrecht, The Netherlands

§ Laboratory of Physical Chemistry, Eindhoven University of Technology, 5612 AZ Eindhoven, The Netherlands

Table of contents

S1 Experimental methods and characterization **2**

S1.1 Materials 2

S1.2 Cadmium myristate precursor 2

S1.3 Absorption spectra products in situ SAXS experiments in homebuilt setup 2

S1.4 Concentration QDs and NPLs from ex situ absorption spectra 3

S1.5 TEM-images of 3.5 ML NPLs, 4.5 ML NPLs and 5.5 ML mini-NPLs 4

S1.6 Tilt series HDAAF-STEM 5.5 ML mini-NPLs 4

S1.7 NPLs synthesis with cesium acetate 5

S1.8 TEM images and absorption spectra cesium acetate experiments 5

S1.9 Melting point other acetate salts 5

S2 Backgrounds, data correction and analysis of SAXS patterns **6**

S2.1 SAXS patterns of the precursors 6

S2.2 SAXS data processing 7

S2.3 Models for the weighted least-square fitting procedure 8

S2.3.1 Qualitative analysis SAXS scattering pattern QDs and NPLs 8

S2.3.2 Quantum dots 8

S2.3.3 Experiments without acetate addition: stacked mini NPLs 9

S2.3.4 Experiments with acetate addition: large rectangular 4.5 ML NPLs 10

S2.4 Fit results SAXS with and without cadmium acetate addition 11

S2.5 Variations fitting procedure SAXS data with cadmium acetate addition 12

S2.6 SAXS data formation mini-NPLs at 190 °C followed with addition cadmium acetate 12

S3 Fit *In situ* absorption synthesis without addition acetate **13**

S4 Comparison SAXS and ICP-OES results **14**

S4.1 Calculation yield from SAXS data 14

S4.2 Yield and concentration synthesis without addition $Cd(Ac)_2$ 14

S4.3 Yield synthesis with addition $Cd(Ac)_2$ 16

S4.4 Preparation and measurement method ICP-OES 16

S1 Experimental methods and characterization

S1.1 Materials

1-butanol (anhydrous, 99.8%), 1-octadecene (ODE, 90%), cadmium(II) acetate anhydrous (99.995%), cadmium(II) nitrate tetrahydrate (99,99%, trace metal basis), cesium(I) acetate (99.999%, trace metal basis), hexane (anhydrous, 95%), methanol (anhydrous, 99,8%), methanol (100%), oleic acid (OA, 90%), sodium myristate (≥99%), toluene (anhydrous, 95%) were all purchased from Sigma Aldrich. Selenium powder (200 mesh, 99.999%) was purchased from Brunschwig Chemie. ODE and OA were degassed at 100°C under vacuum for 3h and other chemicals were used without further purification.

S1.2 Cadmium myristate precursor

Cadmium myristate precursor was prepared by dissolving 1.23 g cadmium nitrate tetrahydrate (4 mmol) in 40 mL methanol and 3.13 g sodium myristate (12.5 mmol) in 250 mL methanol. The cadmium nitrate solution was added to the sodium myristate solution while stirring. A white precipitate was visible. The precipitate was vacuum filtrated using a Buchner flask and rinsed with 1 L of methanol (hydrous). The cadmium myristate was dried overnight under vacuum to remove methanol and water.

S1.3 Absorption spectra products *in situ* SAXS experiments in homebuilt setup

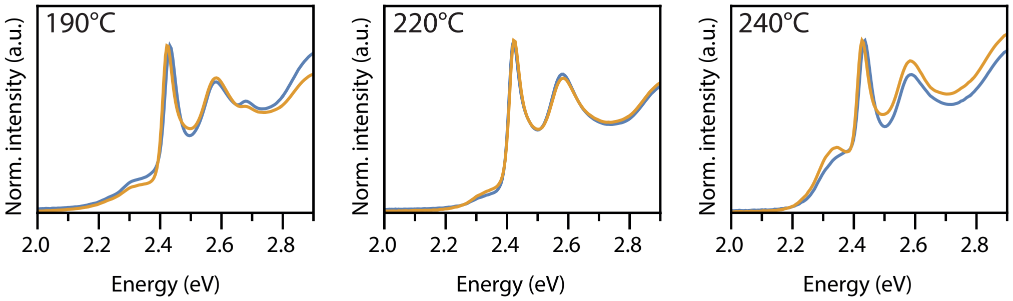

Figure S1 *Ex situ* absorption spectra of the product from *in situ* SAXS experiments where cadmium acetate is added at 190°C, 220°C, and 240°C. Experiments were performed in duplicate (orange and blue). These *ex situ* results verify that synthesis is reproducible in our homebuilt setup. NPLs emitting at 2.7 eV (460 nm) are obtained next to NPLs emitting at 2.45 (510 nm) and QDs at ~2.3 eV (~540 nm) when acetate is added at 190°C. When cadmium acetate is added at 240°C, more QDs compared to NPLs are formed. Addition at 220°C results in the formation of only one population of NPLs and a smaller amount of QDs. For these reasons we used the 220°C procedure in the main text.

S1.4 Concentration QDs and NPLs from *ex situ* absorption spectra

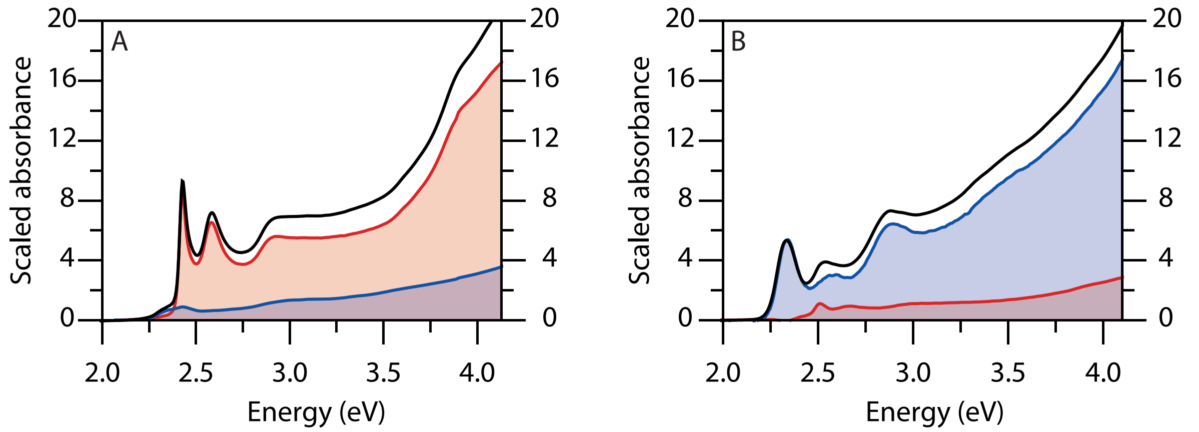

Figure S2 Absorption spectra from main text with absorbance at high energy for determination of the QD and NPL concentration. The absorbance is scaled to match the absorbance of the reaction mixture in the reaction flask at 240°C. (A) Synthesis with addition of cadmium acetate. NPLs (red) and QD (blue) contributions were extracted using size-selective precipitation. (B) Synthesis without addition of cadmium acetate, mini-NPLs and QD contribution are shown in red and blue, respectively. No pure mini-NPL fraction could be extracted, the spectrum in red therefore contains some QD absorption.

The NPL and QD concentration were estimated from the *ex situ* spectra in Figure 1C (S2A) and Figure 4C (S2B) using Lambert-Beers law. The contribution of the NPLs- or QDs absorbance to the total absorbance at 300 nm (4.13 eV) was determined by fitting the spectra with the QD and NPL fractions obtained with size-selective precipitation. The absorption coefficients well above the band edge absorption were calculated for the NPLs and QDs as described in literature.[1-3] For these calculations, the QD volume was extracted from the absorption spectra using the sizing curve by Hens et al.[2] and the volume of the NPLs was obtained from TEM analysis. The resulting concentrations of the product dispersions were scaled to match the volume during the synthesis at 240°C, *i.e.*, scaled to a total volume of 9.4 mL (7.5 mL ODE and a volume expansion of 25% at 240°C.[4]) This ensures that the calculated concentrations can directly be compared to the concentrations obtained from the *in situ* SAXS analysis. A QD and NPL concentration of 2.9 µM and 0.35 µM were obtained for the synthesis with addition of cadmium acetate. A QD concentration of 11.2 µM was extracted for the synthesis without addition cadmium acetate.

S1.5 TEM-images of 3.5 ML NPLs, 4.5 ML NPLs and 5.5 ML mini-NPLs

The TEM-images and HDAAF-STEM images in the SI or main text were either recorded on a FEI Technai 20 operating at 80 keV or a Talos F200X operating at 200 keV. Samples were made by drop-casting a dilute dispersion of nanoparticles on a TEM-grid.

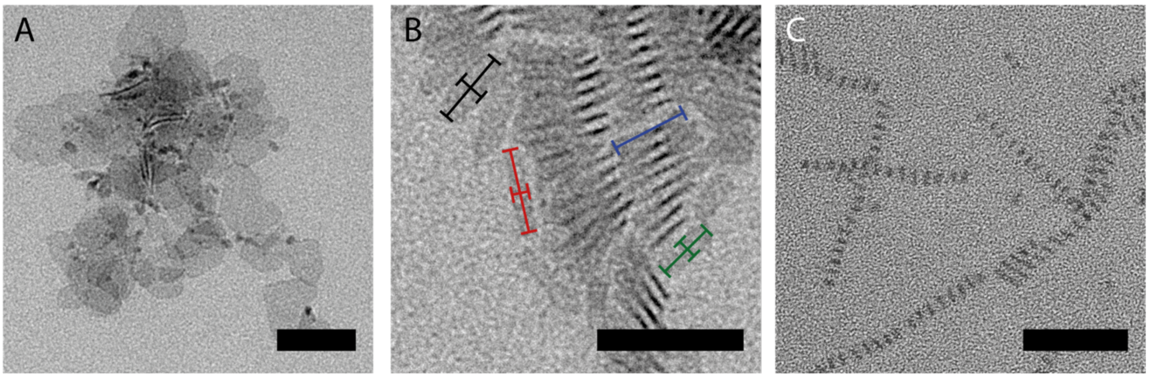

Figure S3 (A) TEM-image displaying 3.5 ML NPLs and QDs obtained when cadmium acetate is added at room temperature and the reaction mixture is heated to 240°C for 10 minutes in a standard three-neck flask. (B) TEM-image of 4.5 ML NPLs obtained during SAXS experiment in homebuilt setup with addition of cadmium acetate at 220°C. The lateral dimensions of a few NPLs are indicated in the image. The average lateral dimensions, 27 ± 2.2 nm by 7.5 ± 1.2 nm, were determined by measuring >100 NPLs. (C) Bright-field TEM image 5.5 ML mini-NPLs obtained when no cadmium acetate is added. Synthesis performed in homebuilt setup. Scalebars correspond to 50 nm.

S1.6 Tilt series HDAAF-STEM 5.5 ML mini-NPLs

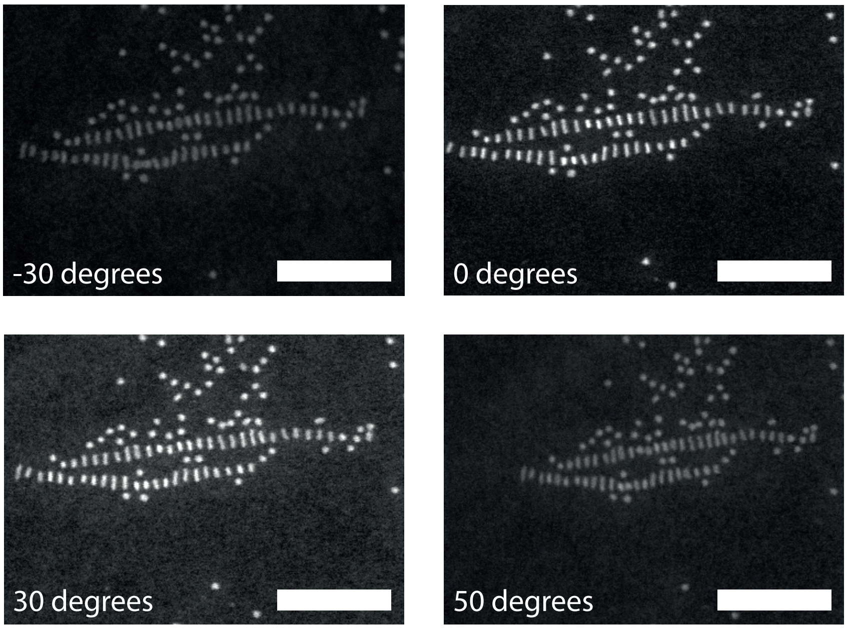

Figure S4 Tilt series of reaction product without addition of cadmium acetate. The HDAAF-STEM images were measured for the reaction product without size-selective precipitation and show the presence of both mini-NPLs and QDs.

S1.7 NPLs synthesis with cesium acetate

The synthesis method for NPLs with the addition of cesium acetate is similar to the method reported in the main text. The amounts were not halved for these experiments and were performed in a standard 100 mL three-neck round-bottom flask. 86 mg cesium acetate (0.44 mmol) was added at 190°C. A twice as high amount in mmol of cesium(I)acetate compared to cadmium(II)acetate was added to ensure that an equal amount of acetate was present during the reaction.

S1.8 TEM images and absorption spectra cesium acetate experiments

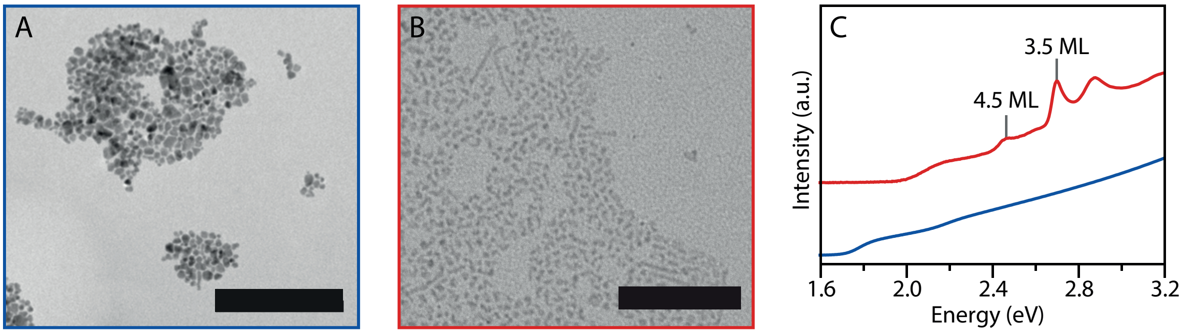

Figure S5 TEM-images of the synthesis product when cesium acetate is added at 190°C and temperature is increased to 240°C, scale bar 200 nm (A) or cesium acetate is added at 190°C without increasing the temperature, scale bar 100 nm (B). The absorption spectra are shown in blue and red, respectively (C). NPLs are only observed on TEM images and in absorption spectra when the temperature is not increased above the melting temperature of cesium acetate (195°C).

S1.9 Melting point other acetate salts

In the literature other acetate salts have been reported, including $Mn(Ac)_2.4H_2O$, $Zn(Ac)_2$, Mg(Ac)2.4H_2O; $Co(Ac)_2.4H_2O$, and Na(Ac), to promote formation of CdSe NPLs.[5] All these mentioned acetates resulted in the formation of NPLs in our own experiments as well (addition temperature of 190°C). The melting temperature of all these acetates is higher than 190°C: $Co(Ac)_2$ (298°C), $Mn(Ac)_2$ (210°C), $Zn(Ac)_2$ (257°C), $Na(Ac)_2$ (324°C). For $Mg(Ac)_2.4H_2O$, a melting point of 90°C is often reported. However, $Mg(Ac)_2$ dissolves in the water incorporated in the crystal at this temperature, giving a seemingly lower melting temperature. A melting temperature of ~230°C is reported by Yu Jiang *et al.* for $Mg(Ac)_2$.[6] In this respect, it is worth noting that we only observe anhydrous cadmium acetate in the scattering patterns (peak at $q = 6.63\ nm^{-1}$) when we either add anhydrous or hydrous cadmium acetate. The water incorporated in the crystal is apparently quickly lost. Therefore, we consider the melting points of the anhydrous salt even if the hydrous version is added.

S2 Backgrounds, data correction and analysis of SAXS patterns

S2.1 SAXS patterns of the precursors

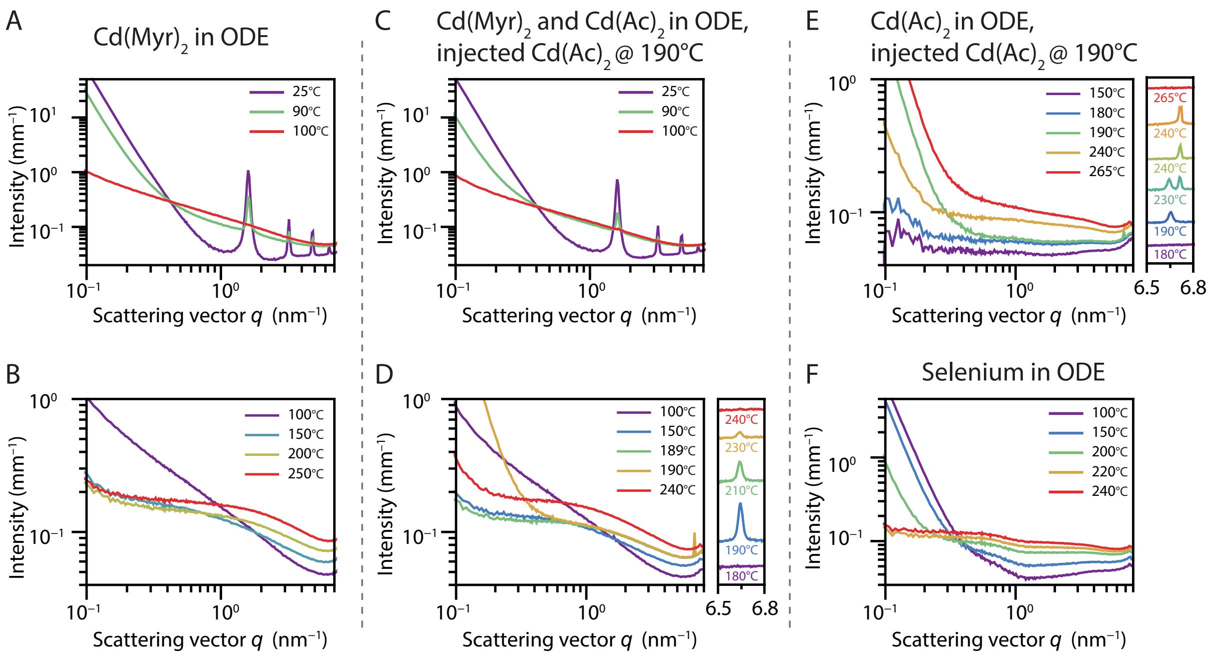

Figure S6 Left: Scattering patterns of cadmium myristate in ODE at low (A) and high (B) temperatures. At low temperatures a lamellar phase is observed, which melts around 100°C. The scattering patterns of experiments discussed in the main text were corrected using these scattering patterns. **Middle:** scattering patterns of cadmium myristate in ODE with the addition of cadmium acetate at 190°C. The addition of cadmium acetate can be observed by the increase in scattering at small q-values ($q < 0.2$ nm^{-1}) and by a peak at ~6.7 nm^{-1} (orange, D). The scattering peak is plotted more clearly next to the bottom image. These scattering patterns are shifted for clarity. The increase in intensity at $q < 0.2$ nm^{-1} and the peak at ~6.7 nm^{-1} disappear upon heating the mixture to 230°C. Note: This temperature is lower compared to when the acetate is injected at 220°C (experiment in the main text) because the acetate is introduced earlier. **Right:** (E) scattering patterns of cadmium acetate in ODE at different temperatures (acetate injected at 190°C). The increase in scattering at small q and the appearance of a peak at ~6.7 nm^{-1} upon addition of the acetate is also observed here. Around 230°C, the peak shifts to higher q-values, similar to what was observed during the experiment in the main text. The cadmium acetate salt does not melt/dissolve at temperatures lower than its melting temperature (255°C) without the presence of cadmium myristate in the mixture. In the right panel, where the cadmium acetate peak is shown, the peak is still observed after prolonged heating at 240°C. The two plots at 240°C are 20 min apart. This indicates that the cadmium acetate reacts with cadmium myristate when both are present in the reaction mixture. (F) Scattering patterns at different temperatures of selenium in ODE (selenium added at room temperature). The presence of large selenium clumps results in scattering at small q-values. Selenium starts to dissolve after reaching 150°C. Selenium in ODE gives an almost constant background at 220°C and higher.

S2.2 SAXS data processing

We obtained the 1D scattering curves of the growing nanocrystals by azimuthal integration of the patterns recorded on the 2D detector. The curves were processed by subtracting the temperature-dependent scattering of a cadmium myristate precursor solution in the reaction flask (Figure S6, left) and the scattering of the empty flask:

$$I_{\mathrm{proc}}(T) = I_{\mathrm{raw}}(T) - aI_{\mathrm{flask}} - b\left[I_{\mathrm{Cd(Myr)_2\ in\ ODE}}(T) - I_{\mathrm{flask}}\right]$$

The values for *a* varied between 0.95 and 1.05. A constant value of ~0.8 was used for *b* for all temperatures. This value was determined at 240°C. After this processing, $I_{\mathrm{proc}}(T)$ still contained some residual cadmium myristate scattering before the temperature reached 240°C because less myristate precursor had reacted. This additional scattering was accounted for during the fit by approximating the scattering, arbitrarily, with the form factor of a sphere with a radius of ~0.7 nm ($P_{\mathrm{sphere,myr}}$ in section S2.3.3 and S2.3.4). Figure S7 shows that the scattering of cadmium myristate resembles that of a sphere (red) for $q > 1$ nm^{-1}. At these *q*-values, cadmium myristate background is the dominant contribution to the scattering signal in the initial stages of our *in situ* synthesis experiments. The strength of this background signal decreases as the reaction mixture is heated and is negligible when the temperature hits 240°C. Selenium does not produce any significant background at intermediate *q*-values, and it fully dissolves in ODE at the temperatures of interest.

The absolute scattering intensity was corrected for the pathlength of X-rays through the reaction flask, which ranged from 3.4 mm to 3.8 mm, and was determined by measuring the scattering intensity of water for each of the different flasks used during our experiments. The scattering intensity could then be calibrated using the known absolute scattering cross-section of water (1.65 10^{-3} mm^{-1} at 16 keV).[7]

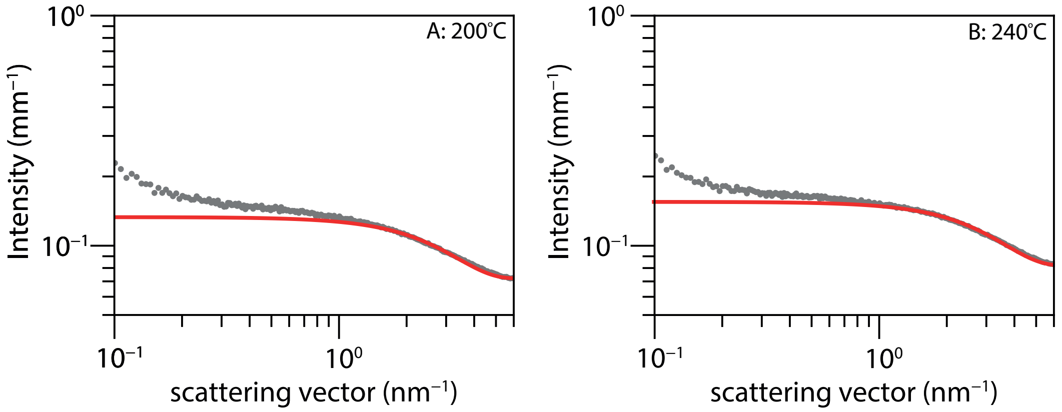

Figure S7 The scattering of cadmium myristate (gray) at 200°C (A) and 240°C (B) can be approximated with the scattering pattern of a spherical particle with a radius of 0.7 nm (red). This calculated scattering curve was used during the fitting procedure to fit the initially higher scattering of cadmium myristate, which was underestimated by the correction described above.

S2.3 Models for the weighted least-square fitting procedure

S2.3.1 Qualitative analysis SAXS scattering pattern QDs and NPLs

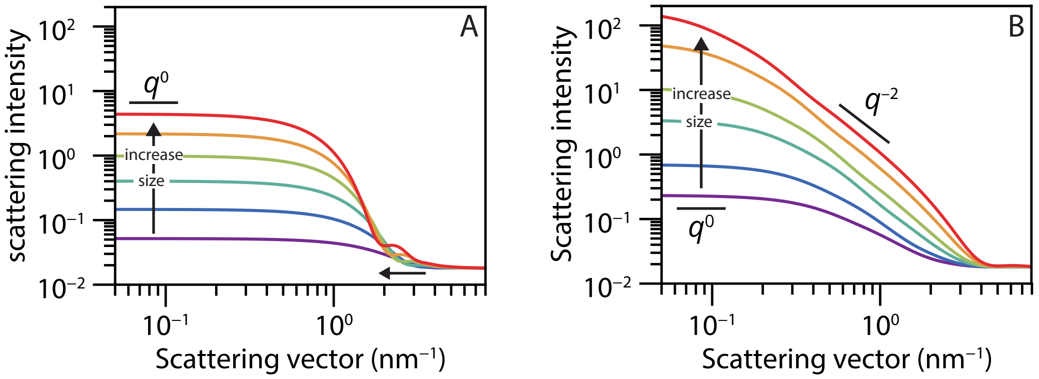

Figure S8 Theoretical form factor of (A) spheres and (B) rectangular 2D particles with a thickness of 1.3 nm. A similar polydispersity of the particles and amount of background scattering are used as in the experiments. The number density of the rectangular particles is one order of magnitude lower than the QDs. QD diameters considered in the panel range from 2 to 4.5 nm. For larger QDs, the minima shift to smaller q values. The lateral dimensions of the NPLs in panel b range from 1.5 nm by 4.5 nm to 8 nm by 24 nm. Qualitatively, NPLs and QDs can easily be distinguished in SAXS; both have a regime where the scattering is independent of q (indicated with q^0), but the NPLs also have a range where the slope scales with q^{-2}. Additionally, the minima for rectangular particles are less pronounced due to rotational averaging.

S2.3.2 Quantum dots

CdSe quantum dots (QDs) have a quasi-spherical shape. Good fit results can be obtained from SAXS data of CdSe QDs by assuming spheres with a Gaussian size distribution. The scattering intensity of spherical particles with a Gaussian distribution of radii is given by

$$I(q) = n_1\, \Delta\rho^2\, \langle P_{\mathrm{sphere}}(q)\rangle_R\, S_{\mathrm{spheres}}(q)$$

where n_1 is the number density, $\Delta\rho$ the scattering length density (SLD) contrast, $\langle P_{\mathrm{sphere}}(q)\rangle_R$ the average form factor for spherical particles with average radius R_0 and standard deviation σ_{R}. $S_{\mathrm{spheres}}(q)$ is the structure factor due to positional order between the particles. In our experiments, the QD density remains low and the inter-QD interactions are weak, so $S_{\mathrm{spheres}}(q) = 1$. The form factor of a sphere of radius R is

$$P_{\mathrm{sphere}}(q) = 36\pi\, \Delta\rho^2\, V_{\mathrm{sphere}}^2 \frac{(\sin qR - qR\cos qR)^2}{(qR)^6}.$$

Averaging over a Gaussian size distribution is described by

$$\langle P_{\mathrm{sphere}}(q)\rangle_R = 36\pi\, \Delta\rho^2 \frac{1}{\sqrt{2\pi}\sigma_R} \int_0^\infty e^{-\frac{1}{2}\left(\frac{R-R_0}{\sigma_{\mathrm{R}}}\right)^2} V_{\mathrm{sphere}}^2 \frac{(\sin qR - qR\cos qR)^2}{(qR)^6} \mathrm{d}R,$$

where V_{sphere} is the volume of a sphere with radius R. $\Delta\rho$ is calculated by subtracting the SLD of the reaction mixture from that of CdSe: $\Delta\rho = \rho_{\mathrm{CdSe}} - \rho_{\mathrm{reaction\ mixture}}$. Using the SLD calculator provided by the National Institute of Standards and Technology (NIST, https://www.ncnr.nist.gov/resourses/activation/) we obtained $\rho_{\mathrm{CdSe}} = 40.6 \times 10^{-6}$ Å^{-1} for CdSe at 16 keV. For the organic reaction mixture, we considered a mass density of 0.63 g cm^{-3} at 240°C[4] due to thermal expansion. With this density, we obtain $\rho_{\mathrm{reaction\ mixture}} = 6.1 \times 10^{-6}$ Å^{-1} at 16 keV.

S2.3.3 Experiments without acetate addition: stacked mini NPLs

The scattering intensity of a stack of NPLs can be approximated by[8]

$$I(q) = n_2\, \Delta\rho^2\, \langle P_{\mathrm{NPLs}}(q)\rangle_R\, F_{\mathrm{stacking}}(q)$$

where n_2 is the number density of the NPLs, $\Delta\rho$ the SLD contrast between the NPL and the reaction mixture (see S2.3.2), $\langle P_{\mathrm{NPLs}}(q)\rangle_R$ the average form factor of the lateral shape of the NPLs and $F_{\mathrm{stacking}}(q)$ the shape factor for the structure in the perpendicular direction. The mini-NPLs were, for simplicity, fitted with the form factor of a disk with radius R:

$$P_{\mathrm{mini-NPL}}(q) = 16\pi\, \Delta\rho^2\, A_{\mathrm{NPL}}^2 \frac{2J_1(qR)^2}{(qR)^2}$$

With a Gaussian distribution in the radii of the disk, this becomes

$$\langle P_{\mathrm{mini-NPLs}}(q)\rangle_R = 16\pi\, \Delta\rho^2 \frac{1}{\sqrt{2\pi}\sigma_{\mathrm{R}}} \int_0^\infty e^{-\frac{1}{2}\left(\frac{R-R_0}{\sigma_{\mathrm{R}}}\right)^2} A_{\mathrm{NPL}}^2 \frac{2J_1(qR)^2}{(qR)^2}\, \mathrm{d}R,$$

with A_{NPL} the area of a disk with radius R, $J_1(qR)$ the first-order Bessel function, R_0 the average radius and σ_{R} the standard deviation in R.

The shape factor $F_{\mathrm{stacking}}(q)$ is the Fourier transform of the electron density profile in the stacking direction and is given by[9]

$$F_{\mathrm{stacking}}(q) = NL^2 \frac{\sin(qL/2)}{qL/2} \frac{\sin(qNd/2)}{\sin(qd/2)}$$

where L is the thickness of the 5.5 ML mini-NPLs which is 1.6 nm, d the spacing between two NPLs which could be determined by the position of the structure factor peaks (d = 4.5 nm), N is the number of NPLs in a stack. A Gaussian distribution was also used for the number of NPLs in a stack:

$$\left\langle F_{\mathrm{stacking}}(q)\right\rangle_N = \frac{L^2}{\sqrt{2\pi}\sigma_{\mathrm{N}}} \int_0^\infty e^{-\frac{1}{2}\left(\frac{N-N_0}{\sigma_N}\right)^2} N \frac{\sin(qL/2)}{qL/2} \frac{\sin(qNd/2)}{\sin(qd/2)}\, \mathrm{d}N$$

The complete model, used for the synthesis where no acetate is added, is:

$$I(q) = B + n_0\, P_{\mathrm{sphere,myr}}(q) + n_1\, \Delta\rho^2\, \langle P_{\mathrm{sphere}}(q)\rangle_R + n_2\, \Delta\rho^2\, \langle P_{\mathrm{mini-NPLs}}(q)\rangle_R \left\langle F_{\mathrm{stacking}}(q)\right\rangle_N$$

The first term is the background scattering and the second accounts for the additional scattering of cadmium myristate during the heat-up. The scattering patterns were fitted using this model, with B, n_0, n_1, n_2, $R_{\mathrm{mini-NPLs}}$, R_{QDs}, $\sigma_{\mathrm{R,mini-NPLs}}$, $\sigma_{\mathrm{R,QDs}}$, N and σ_{N} as free fit parameters. An example of a fit with the contribution of the QDs and stacked NPLs is shown in Figure S9

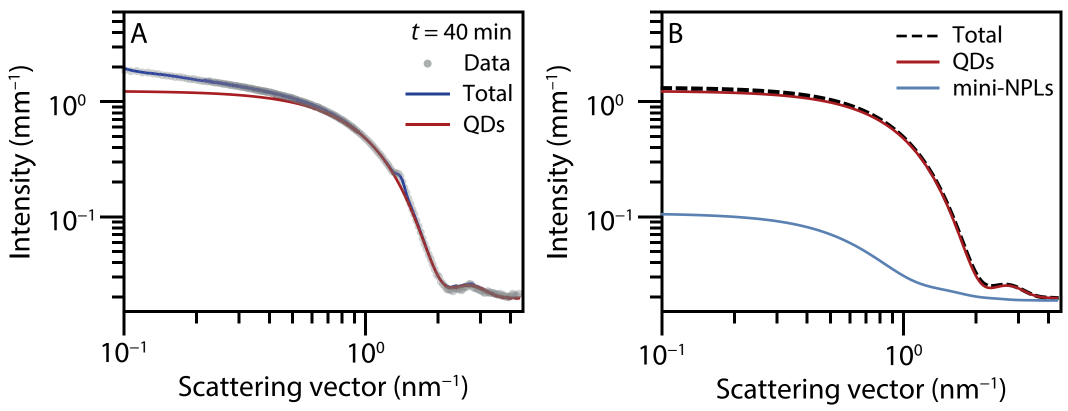

Figure S9 (A) Fit (blue) of scattering pattern 40 min after reaching a temperature of 220℃ using a model for scattering of QDs and stacked 5.5 ML mini-NPLs described in section S2.3.1 and S2.3.2. The contribution of the QDs is shown in red. (B) Scattering intensity of unstacked 5.5 ML mini-NPLs compared to the fitted QD-scattering in (A). In blue the scattering of 5.5 ML mini-NPLs with a 3.5 nm radius, thickness of 1.6 nm, and a concentration of 0.25 µM. These values were extracted from the fit in (A). The effect of unstacked mini-NPLs on the total scattering (black dashed) is neglectable. The same holds for NPLs with small lateral dimensions in a synthesis with addition of cadmium acetate. The increase in scattering at small *q* in (A) is mainly caused by the stacks of mini-NPLs, not by the scattering of individual mini-NPLs.

S2.3.4 Experiments with acetate addition: large rectangular 4.5 ML NPLs

The large 4.5 ML NPLs obtained when cadmium acetate is added during the reaction could not be fitted well with the form factor of a disk (in contrast to the 5.5 ML mini-NPLs). These scattering patterns were fitted using the form factor of a rectangular-shaped particle with a thickness of 1.3 nm, corresponding to 4.5 ML NPLs. The form factor, averaged over all orientations, for this anisotropic particle is given by[10]

$$P_{\mathrm{NPL}}(q)=\int_0^{2\pi}\int_0^{\pi}\left|4RWd\,\frac{\sin(q_xR)}{q_xR}\,\frac{\sin(q_yW)}{q_yW}\,\frac{\sin(q_zd/2)}{q_zd/2}\right|^2\sin\theta\,\mathrm{d}\theta\,\mathrm{d}\phi$$

$$q_x=q\sin\theta\cos\phi$$
$$q_y=q\sin\theta\sin\phi$$
$$q_z=q\cos\theta$$

where R, W and d are the half-length, half-width and thickness of the particles, respectively. Best fits were obtained with W equal to $3R$. Again, a Gaussian distribution in R was used.

The complete model to fit the scattering data for experiments with acetate addition is

$$I(q)=B+n_0\,P_{\mathrm{sphere,myr}}(q)+n_1\,\Delta\rho^2\,\langle P_{\mathrm{sphere}}(q)\rangle_R+n_2\,\Delta\rho^2\,\langle P_{\mathrm{NPLs}}(q)\rangle_R+aI_{\mathrm{Cd(Ac)_2\ in\ ODE}}$$

The first term accounts for background scattering and the second for the additional myristate scattering during the heat-up. The last term accounts for the scattering of the cadmium acetate crystallites in the reaction mixture. The ratio between the lateral dimension, $W/R=3$, and the NPL concentration, $n_2/N_{\mathrm{A}}=c_{\mathrm{NPL}}=0.6\ \mathrm{\mu M}$, were determined from a fit to the scattering curve at an experiment time where the cadmium acetate has melted, i.e., $t=9$ min. The values were fixed for the fits to the curves at all other times.

Both experiments (with or without acetate addition) saw the polydispersity of the QDs ($\sigma_{\mathrm{R,QDs}}$) increase to a value of 0.5 nm during the heat-up to 220°C. Background scattering from cadmium acetate, following addition at 220°C, complicated reliable fitting of the scattering contribution from QDs. We therefore fixed the QD polydispersity at $\sigma_{R,\mathrm{QDs}} = 0.5\ \mathrm{nm}$ for the analysis of all scattering curves following acetate addition. Examples of fitted data before and after cadmium acetate dissolution are shown in Figure S10.

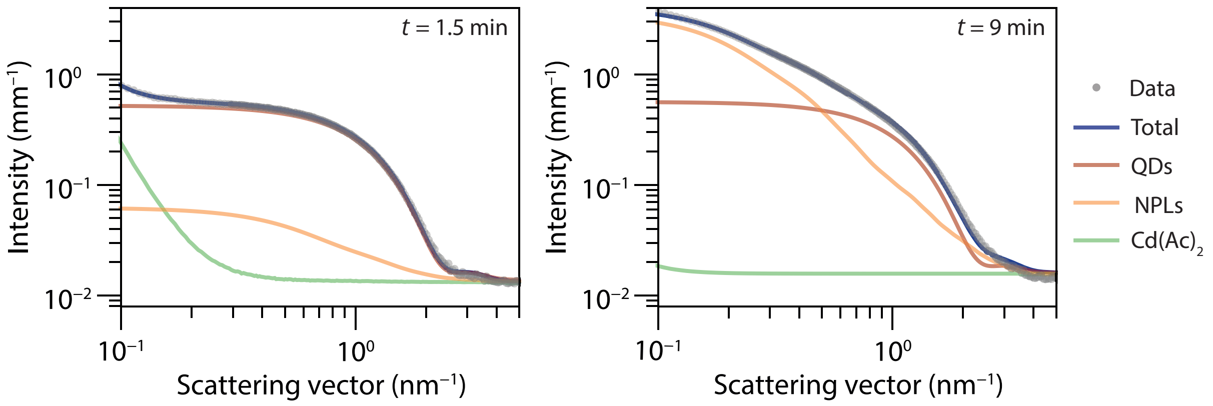

Figure S10 Fitted scattering patterns at t = 1.5 min (left) and t = 9 min (right) with the contribution of QD-, NPL- and cadmium acetate scattering to the total scattering. The fitted intensity of the flat background scattering B (~0.015 mm^{-1}) is added to the fits shown in blue, red, orange, and green.

S2.4 Fit results SAXS with and without cadmium acetate addition

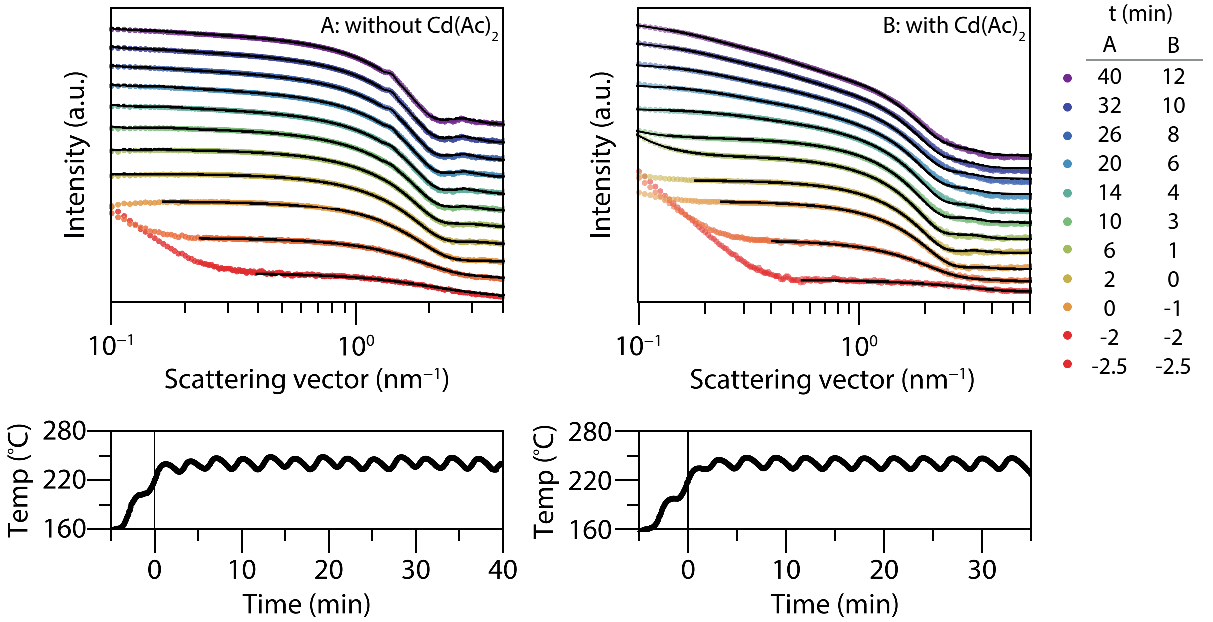

Figure S11 Selection of scattering patterns with fit (black) obtained using the models described above. Synthesis (A) without and (B) with the addition of cadmium acetate at t = 0. Scattering patterns are shifted in intensity for clarity. Below the temperature profiles are given of the respective experiments.

S2.5 Variations fitting procedure SAXS data with cadmium acetate addition

In Figure S12, we check that our fit results are robust to slight variations of the model used. We compare the fit results obtained with a fixed time-independent NPL concentration c_{NPL} to those obtained when c_{NPL} is a free parameter, and the results obtained with and without the inclusion of a background contribution due to cadmium acetate. The estimated lateral dimensions are similar at t > 7.5 min for three variations considered to the model (blue, red, and green). The variation where both the NPLs concentration and cadmium acetate background was fitted (orange) gives slightly smaller values because it overestimates the cadmium acetate background. However, if no cadmium acetate background is included in the model, the NPL concentration shows an unrealistic peak in the NPL concentration around t = 2 min and the lateral dimensions are very large at t < 2.5 min (Figure S12, green). This is likely an artifact due to the false assignment of the sloping background scattering of cadmium acetate to NPLs. We resolve this difficulty by fixing c_{NPL} = 0.6 µM, which is the constant value extracted for t > 5 min (Figure S12B). This might give rise to a slight overestimation of the lateral dimension in the first minutes after the addition of cadmium acetate. The value of 0.6 µM is similar to the concentration of mini-NPLs obtained when no acetate is added and therefore is likely a good estimate of the NPL concentration around t = 0–5 min.

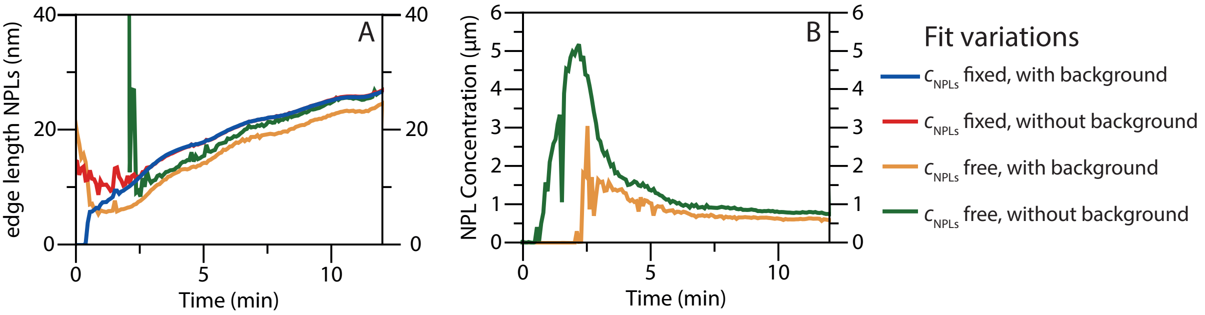

Figure S12 Fit results of the synthesis with cadmium acetate addition (Figure S11B) using four variations to the model described in section S2.3.4: (A) the NPL size, expressed in terms of the longest edge length while the aspect ratio is fixed at 3:1, and (B) NPL concentration. We kept the NPL concentration fixed at c_{NPL} = 0.6 µM (blue and red) or used it as a free fit parameter (orange and green). We did (blue and orange) or did not (red and green) include a background contribution of Cd-acetate.

S2.6 SAXS data formation mini-NPLs at 190°C followed with addition cadmium acetate

The data in the main text shows that 5.5 ML mini-NPLs form when the reaction mixture is heated to 240°C without adding cadmium acetate at 190°C. Thinner mini-NPLs can be synthesized when the reaction temperature is kept at 190°C instead. The structure factor peaks in Figure S13A show that 4.5 ML mini-NPLs form after prolonged heating at 190°C. Large 4.5 ML NPLs can still be formed when cadmium acetate is added after 35 min at 190°C. The reaction temperature is also increased to 240°C as for a standard 4.5ML NPLs synthesis (Figure S13B).

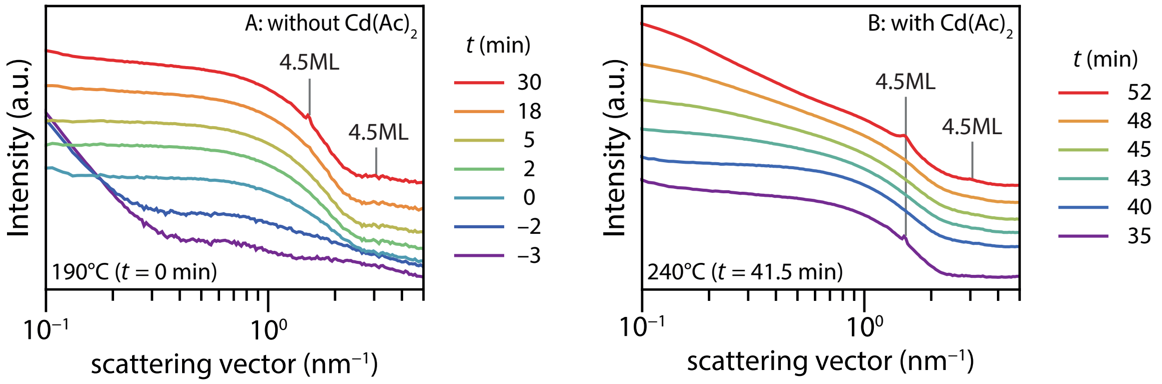

Figure S13 (A) Scattering data without adding cadmium acetate with a reaction temperature of 190°C instead of 240°C as in the main text. The scattering data corresponds to the form factor of spherical NCs, but after prolonged heating, structure factor peaks appear corresponding to 4.5ML mini-NPLs (red). (B) Upon addition of cadmium acetate (t = 35 min), the mini-NPLs first unstack (purple to blue spectrum), and the lateral dimension of the NPLs increase (green–orange), which can be deduced from the change in slope for $q < 1$ nm^{-1}. At the end of the reaction, the large NPLs also form stacks, indicated by the structure factor peaks. The peaks' positions correspond to 4.5 ML NPLs.

S3 Fit *In situ* absorption synthesis without addition acetate

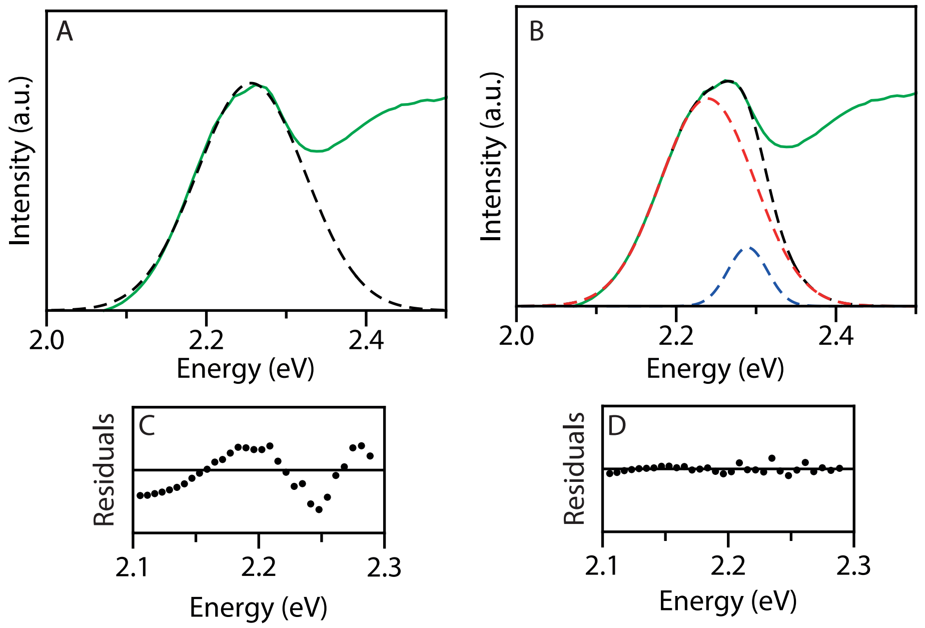

Figure S14 Single gaussian (A) and double Gaussian (B) fit of first exciton transition in the *in situ* absorption spectrum at t = 10 min and T = 240°C for the synthesis without addition of cadmium acetate (orange spectrum figure 4A). The fit and the fit residuals of the single Gaussian fit (A and C) show a systematic deviation and reveal an additional feature on the high energy side. The double Gaussian fits the data well (see fit residuals, D) and reveals a small extra absorption peak at the position of the 5.5 ML mini-NPLs (at ~2.28 eV).

S4 Comparison SAXS and ICP-OES results

S4.1 Calculation yield from SAXS data

The yields of the reaction displayed in Figures 3C and 4F were determined from the SAXS data by calculating the amount of selenium incorporated into the QDs and (mini-)NPLs and the amount of selenium added at the start of the reaction (6 mg) because selenium is the element in deficit. From the SAXS scattering patterns, we extracted the molar concentration ($n_{\mathrm{NP}}/N_{\mathrm{A}} = c_{\mathrm{NP}}$) and average volume $\langle V_{\mathrm{NP}} \rangle_R$ of the nanoparticles (NPs), where R are the radii (QDs and mini-NPLs) or edge lengths (NPLs). The yield can be obtained by calculating the average amount of selenium in a NP

$$N_{Se} = \frac{4\langle V_{NP} \rangle_R}{a^3}$$

where $a^3 = 0.22\, nm^3$ is the volume of the cubic unit cell of zinc blende CdSe. The factor 4 comes in because a unit cell contains four Se atoms. The yield can then be expressed as:

$$Yield\ (\%) = c_{NC} \frac{N_{Se}}{c_0} \cdot 100\%,$$

where c_0 is the initial concentration of selenium ($8.2 \cdot 10^{-3}$ M), i.e., 6 mg in a total reaction volume of 9.4 mL, taking the thermal expansion of the reaction mixture into account.
Note that for the volume of the NPLs and mini-NPLs a thickness of 1.2 nm and 1.5 nm is used instead of the actual thickness (1.3 nm and 1.6 nm) to account for the additional cadmium layer. The number of selenium atoms in a (mini-)NPL would be overestimated without this adjustment.

S4.2 Yield and concentration synthesis without addition $Cd(Ac)_2$

Figure 4F of the main text shows the CdSe consumption by the QDs (CdSe yield by QDs) when no cadmium acetate is added. For the total yield of the synthesis also the CdSe consumption by the mini-NPLs should be considered. The concentration and size of the mini-NPLs could be extracted from the SAXS data when the mini-NPLs started to stack. The reaction yield as a function of reaction time was then calculated as

$$Yield\ (\%) = \frac{4\langle V_{mini-NPL} \rangle_R}{a^3} \frac{c_{mini-NPLs}}{c_0} \cdot 100\%,$$

where $\langle V_{NPL} \rangle_R$ is the NPL volume averaged over the fitted distribution of disk radii, $a^3 = 0.22\, nm^3$ is the volume of the cubic unit cell of zinc blende CdSe, and c_0 is the initial concentration of Se (the element in deficit) in the reaction mixture. The factor 4 comes in because a unit cell contains four Se atoms. To calculated the volume, relevant for Se consumption, a thickness of 1.5 nm instead of 1.6 nm was used to compensate for the additional cadmium layer as both top and bottom facets are Cd-terminated.
The concentration and yield of the stacked mini-NPLs are shown in Figure S15. After 30 – 40 min, the concentration was ~0.25 µM and the yield ~3%. This results in a total yield, QD and mini-NPLs CdSe consumption of 81% after a reaction time of 40 min.
The concentration and yield of the mini-NPLs could be underestimated because only the concentration of the stacked mini-NPLs could be extracted from the SAXS data. Therefore, the total reaction yield was also determined *ex-situ* with ICP-OES. The selenium concentration (element in deficit) in a sample cannot be determined directly with ICP because sample preparation with an acid results in the formation of H_2Se, which escapes. Instead, the cadmium concentration was measured and a molar ratio of Se:Cd = 1 in the QDs and mini-NPLs was assumed to determine the yield of the reaction. The sample preparation is discussed in the following section. The yields obtained after a reaction time of 10 min and 30 min are shown in Table 1 in Figure S15.

The yield obtained via ICP-OES is similar to the SAXS results. After 30 min, the total yield is ~75% and ~79%, respectively. After 13 min, the yield is a bit lower in the ICP analysis (~47% compared to ~72%), which is likely due to the heat-up difference in a standard three-neck flask compared to the flask used for SAXS analysis. Overall, the comparison of ICP and SAXS results shows that the mini-NPL concentration and yield are not heavily underestimated with only probing the stacked mini-NPLs.

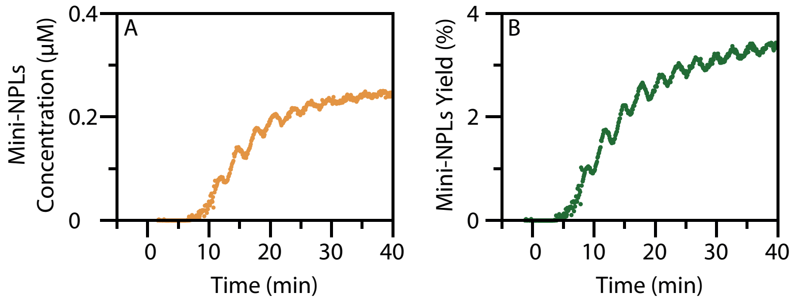

Table 1: Yield ICP-OES and SAXS without addition $Cd(Ac)_2$

ICP-OES		SAXS			
t (min)	Total Yield (%)	*t* (min)	Total Yield (%)	QD Yield (%)	mini-NPL Yield (%)
13	52 ± 0.8	13	71.5 – 73	70	1.5 – 3
13	44 ± 0.8	30	79	76	3
13	42 ± 2.7	40	81	78	3
13	49 ± 0.9				
30	74 ± 1.0				
30	75 ± 0.9				

Figure S15 Concentration (A) and yield (B) of 5.5 ML mini-NPLs as a function of reaction time for synthesis without addition cadmium acetate. The oscillations are caused by temperature oscillation (S2.4) affecting the expansion of the reaction flask and reaction volume. The concentration and yield plotted are underestimated values because only the scattering of stacking mini-NPLs is visible over scattering of QDs, while scattering of individual mini-NPLs is too weak. The growth of mini-NPL concentration and yield between t = 5 min and 30 min reflect that more and longer stacks are formed. Starting from t = 30 min, the NPL concentration levels off to a constant value of 0.25 μM. Table 1 shows the total yield determined with ICP-OES and SAXS for reactions without addition of cadmium acetate. The values represent the selenium consumption by QD and mini-NPLs. At 13 min, there is not much stacking of the mini-NPLs yet. The yield is estimated to be between 1.5% and 3%.

S4.3 Yield synthesis with addition $Cd(Ac)_2$

The SAXS yield was also verified with ICP-OES for the synthesis with addition of cadmium acetate. The comparison is shown below in Table 2. The ICP yield was measured thrice. For the ICP results, a molar ratio of Se:Cd = 1 in the QDs and NPLs was used. Due to the additional cadmium layer, this ratio will deviate for the NPLs. Also here, the yield around 13 minutes is slightly lower, likely due the heat-up difference in a standard three-neck flask compared to the flask used for SAXS analysis. Both the SAXS and ICP results show that at 13 min a higher total reaction yield is obtained when cadmium acetate is added to the reaction mixture.

Table 2: Yield ICP-OES and SAXS with addition $Cd(Ac)_2$

ICP-OES		SAXS			
t (min)	Total Yield (%)	*t* (min)	Total Yield (%)	QD Yield (%)	NPL Yield (%)
13	63 ± 1.4	12	88	50	38
13	76 ± 1.5				
13	70 ± 1.0				

S4.4 Preparation and measurement method ICP-OES

To prepare the ICP samples, first, the unreacted precursors were removed after a synthesis using the standard washing method discussed in the experimental section. The total mass of the product in hexane was weighted (~10 g). 2 grams of the whole product was taken and put under vacuum to remove hexane. 2 mL 65% nitric acid was added to dissolve the CdSe nanoparticles. After the solution was clear, it was diluted with Milli-Q water to a total volume of 10 mL. The samples were diluted at least 500 times with a 5% nitric acid in milli-Q water solution to obtain a cadmium concentration of ~0.5 ppm. The exact cadmium concentration was determined with a 0 – 1 ppm cadmium in a 5% nitric acid calibration curve made using standard solution 3 of PerkinElmer. Measurements were conducted with a PerkinElmer ICP-OES Optima 8300. The cadmium concentration was measured using the emission lines at 228.8 nm, 214.4 nm, and 226.5 nm. The yield was calculated using the average ppm values obtained with measurements on these three emission lines.

References

1. Achtstein, A. W.; Antanovich, A.; Prudnikau, A.; Scott, R.; Woggon, U.; Artemyev, M., Linear Absorption in CdSe Nanoplates: Thickness and Lateral Size Dependency of the Intrinsic Absorption. *The Journal of Physical Chemistry C* **2015,** *119* (34), 20156-20161.
2. Maes, J.; Castro, N.; De Nolf, K.; Walravens, W.; Abécassis, B.; Hens, Z., Size and Concentration Determination of Colloidal Nanocrystals by Small-Angle X-ray Scattering. *Chemistry of Materials* **2018,** *30* (12), 3952-3962.
3. Hens, Z.; Moreels, I., Light absorption by colloidal semiconductor quantum dots. *Journal of Materials Chemistry* **2012,** *22* (21), 10406-10415.
4. Huang, D.; Simon, S. L.; McKenna, G. B., Chain length dependence of the thermodynamic properties of linear and cyclic alkanes and polymers. *The Journal of Chemical Physics* **2005,** *122* (8), 084907.
5. Ithurria, S.; Dubertret, B., Quasi 2D Colloidal CdSe Platelets with Thicknesses Controlled at the Atomic Level. **2008,** *130* (49), 16504-16505.
6. Jiang, Y.; Chen, H.; Mu, X.; He, Z., Thermal decomposition of magnesium acetate in nitrogen. *Journal of Physics: Conference Series* **2020,** *1653* (1), 012057.
7. Dreiss, C. A.; Jack, K. S.; Parker, A. P., On the absolute calibration of bench-top small-angle X-ray scattering instruments: a comparison of different standard methods. *Journal of Applied Crystallography* **2006,** *39* (1), 32-38.
8. Platz, G., P. Lindner, Th. Zemb (Eds.): Neutron, X-Ray and Light-Scattering: Introduction to an Investigative Tool for Colloidal and Polymer Systems, North Holland, Amsterdam, 1991, ISBN 0-444-88946-9, 375 Seiten, Preis: Dfl. 175. *Berichte der Bunsengesellschaft für physikalische Chemie* **1992,** *96* (6), 839-839.
9. Van Der Stam, W.; Rabouw, F. T.; Geuchies, J. J.; Berends, A. C.; Hinterding, S. O. M.; Geitenbeek, R. G.; Van Der Lit, J.; Prévost, S.; Petukhov, A. V.; De Mello Donega, C., In Situ Probing of Stack-Templated Growth of Ultrathin Cu2–xS Nanosheets. *Chemistry of Materials* **2016,** *28* (17), 6381-6389.
10. Renaud, G.; Lazzari, R.; Leroy, F., Probing surface and interface morphology with Grazing Incidence Small Angle X-Ray Scattering. *Surface Science Reports* **2009,** *64* (8), 255-380.
